# Historical ethnobotanical review of medicinal plants used to treat children diseases in Romania (1860s–1970s)

**DOI:** 10.1186/s13002-020-00364-6

**Published:** 2020-03-24

**Authors:** Madalina Petran, Dorin Dragos, Marilena Gilca

**Affiliations:** 1 Department of Functional Sciences I- Biochemistry, Faculty of Medicine, Carol Davila University of Medicine and Pharmacy, Bucharest, Romania; 2 Nephrology Clinic, University Emergency Hospital Bucharest, 050098 Bucharest, Romania; 3 Department of Medical Semiology, Faculty of Medicine, Carol Davila University of Medicine and Pharmacy, Bucharest, Romania

**Keywords:** Medicinal plants, Ethnopharmacology, Pediatry, Ethnopediatry, Children, Romania

## Abstract

**Background:**

Romanian ethnopediatrics has a long history of medicinal plant use. The main objective of the present review was to identify, collect, systematize, and prioritize the available bibliographical data related to medicinal plants traditionally used to treat various pediatric diseases in Romania during the 1860s–1970s.

**Methods:**

Information was mainly obtained by manual systematic search in various relevant historical works focused on the traditional use of medicinal plants in Romania (1860s–1970s), found in the Archives of Romanian Academy Library and National Romanian Library.

**Results:**

A total of 153 medicinal plants belonging to 52 families were identified as having ethnopediatric significance. The plant traditional indications, targeted body systems, parts used, and way of administration were provided. We have also proposed one index (expressed as percentage) in order to assess the ethnopediatric applicability area of species: ethnopediatric relative therapeutic versatility (ERTV), which was calculated on the basis of the number of distinct uses mentioned for a species. The species identified to have the highest ERTV scores were *Dryopteris filix-mas* (100%), *Gratiola officinalis* (85.71%), *Allium sativum* (71.42%), *Eryngium planum* (71.42%), *Juglans regia* (71.42%), *Matricaria chamomilla* (71.42%), *Plantago major* (71.42%).

**Conclusions:**

The present study exposed for the first time to the international scientific community important ethnopediatric information contained in several local Romanian bibliographical resources that could guide the local and international researchers towards new directions of plant valorization.

## Introduction

### Romanian ethnomedicine and ethnopediatry in the European context—past and present

The majority of the European ethnopharmacological knowledge has its roots in the Greek and Roman cultures, being essentially influenced by works of Dioscorides, Pliny the Elder, Galen, Theophrastus, and Hippocrates [1–3]. Scientists revealed a remarkably consistency between Dioscorides’s *De materia medica* and later European pharmacopeia, Dioscoridean tradition lasting through the nineteenth century with only insignificant variation [4], or even up to the birth of modern pharmacology [2, 3]. While in the Western and Central Europe, herbalism played an important role in drug discovery beginning with the nineteenth century [5]; in Eastern Europe, the exploitation of the ethnopharmacological tradition was hindered by several challenges:

- Vicissitudes related to the social-political-economic environment in those countries belonging to the previous communist bloc, including Romania (e.g., marginalization of experts originating from bourgeois families, and therefore considered “enemies of the regimen,” informational censorship, lack of interest in supporting scientific research and preservation of national cultural heritage, lack of financial support for libraries—Romanian Academy Library was considered too “bourgeois” by the communist regimen, and therefore was replaced in 1955 by State Central Library) [6].
- Other types of challenges: inaccessibility of many of the texts on medicinal plants preserved in locations with limited access to the public (e.g., monasteries with a strict daily schedule, archives not open for the public), reduced legibility of certain manuscripts (lost parts, deterioration in time, low quality of print or indecipherable handwriting), some texts are written in old languages and are not yet translated (e.g., many Romanian manuscripts found in the National Archives are written in old Slavonic language) or investigated by an interdisciplinary team (historians, linguists, anthropologists, botanists, etc.), old terminology with obscure meaning for the contemporary scientist, lack of certainty about the identity of taxa (e.g., only vernacular names or obsolete Latin names), difficulties of communication with informants (e.g., monks who are not allowed to take in face-to-face interviews, suspiciousness of informants caused by superstitions, or desire of the respondents to protect the “secrets” of their medical knowledge, sometimes transmitted only within the families) [4, 7–11].

Some of these aspects might have contributed to the fact that the ethnomedical knowledge of Eastern European countries remained in a certain degree localized, and isolated from the rest of the world [5, 12]. On the other side, due to this isolation and other factors as well (e.g., slower economical development, preservation of small-scale agro-pastoral activities as main economical subsistence tool in rural area [13], continuity of traditional practices in monastic communities, known for their principles of living in harmony with the environment), this part of Europe may still possess a tremendous reservoir of traditional ethnomedical knowledge [13, 14]. For instance, many Romanian Christian Orthodox monasteries are located in the forests, or faraway from inhabited areas, and cultivate principles similar to those of environmentalist sustainability (e.g., respect for Creation/Nature as a manifestation of God, protection of natural resources (gifts of Creation) for future generations, vegetarianism, stability and discipline, etc.) [15]. The monastic community from the Vânători-Neamţ Natural Park, which is recognized as the second largest in Europe, after that of Mt. Athos in Greece, is such an example [15, 16].

It is also known that prior to the twentieth century, European medicine was based mainly on Mediterranean plant-derived drugs (with some additions from the Middle East, Asia, and the Americas) [4], the ethnopharmacological tradition originating in the rest of Europe, such as Eastern European countries including Romania, being underexploited at that time, as well as nowadays.

Many scientists claim that exploring historical texts in a systematic manner may represent a valuable source of knowledge for the rediscovery of forgotten remedies and the development of modern ethnopharmacology [2, 4, 17, 18].

While in some of the Eastern European countries, such as Poland [19–22], Estonia [12, 23, 24], Bosnia and Herzegovina [25, 26], and Russia [27, 28], the existing ethnobotanical resources are already thoroughly studied or actualized in several ethnobotanical surveys; a different situation exists in Romania. The content of the available Romanian ethnographic resources is still unknown by the rest of the world as they are not yet published (e.g., manuscripts found in various museums or libraries), or published only in the national language (e.g., “Botanica Poporana Romana” by Simion Florea Marian). These written resources cover mainly the period until de mid twentieth century.

Romania has a rich ethnomedical and ethnobotanical heritage. According to various antique texts authored by Dioscoride (*Codex Constantinopolitanus*, *De Materia Medica*) and Pseudo Apuleius (*De Herbarum Virtutibus*), many plants (e.g., *Thymus vulgaris*, *Urtica dioica*, *Achillea millefolium*, *Mentha piperita*) have been used as remedies on this territory since millennia, and are still used for similar therapeutic purposes in nowaday ethnomedical practices [29, 30]. Local historical documents mention the art of healing through herbs practiced by indigenous population beginning with the fourteenth century. Several manuscripts originating from various regions of the present day Romania or interwar Romania, dating from the fourteenth–seventeenth centuries, contain elements of medical botany. Some are kept in the Archives of the Library of Romanian Academy (e.g., manuscripts 740, 498, 573, 312) [8], others are found today outside Romania (e.g., the Slavonic manuscript from Hodros-Bodrog monastery, Banat, Romania, written in the fourteenth or fifteenth century, known as *Hodoski sbornic* or *Miscelaneul de la Praga*, is found now in Prague) [31]. The sixteenth century manuscript no.740 in the Romanian Academy Library is considered the oldest text of medical folklore presently existing in Romania. The initial section is entitled “The use of healing plants” and contains 12 pages with descriptions of indications of several medicinal plants (the section was longer, but some pages were lost) (e.g., small and great plantain, angelica, mint, burdock, gentian, etc.). In order to understand the cultural context of our country during the Middle Ages, it should be reminded that the territories of the present day Romania lie within the !!influence area of the Byzantino-Slavic culture; therefore, several of these manuscripts have mixed influences. For instance, despite the fact that manuscript no. 740 is written in Ukrainean Slavic language, it contains influences from Western and Central Europe (probably Dioscoride’s Materia Medica or Matthiolus’s commentaries to Dioscoride’s work), as well as elements of local medical folklore [32].

More systematic documentation of local traditions of plant use in Romania, based on field studies, began in the nineteenth century, with the work of various ethnographers. Simion Florea Marian (1847–1907) initiated this type of work, and he was followed by Nicolae Leon (1862–1931), Charles Laugier (1875–1930), Alexandru Borza (1887–1971), George Bujorean (1893–1971), Valer Butura (1910–1989), and others.

The Romanian ethnopharmacological knowledge and practices were kept alive through oral tradition, within families of healers, midwives, medicinal plant collectors, and monastic communities in a form almost institutionalized until the XXth century [11, 30].

The ethnomedical Romanian practices are also attested by the lexical background. The manuscript *Dictiones latinae cum valachica interpretatione* by Teodor Corbea, the first encyclopedic lexicographic work created in the Romanian space, dating around 1701 and published only recently [33], contains approximately 400 names of plants, including medicinal plants [34]. In 1783, the calvinist priest József Benkő (1740–1814) from Transylvania, published the botanical dictionary “*Nomina vegetabilium*,” which contains 429 species with 612 popular Romanian plants names, some also from the area of !! Muntenia [35]. In other important botanical work, *Transsilvania Generalis*, József Benkő stated that “the Romanian women use efficiently weeds neglected by others, to cure various diseases” [35].

Despite this valuable biocultural heritage, only a few local teams of researchers focused their attention on Romanian ethnopharmacological practices in the last decades [36–41]. The data collected by Romanian scientists in field studies after 1970s have been reflected in only a few reliable scientific publications [38, 39, 41]. We have also noticed a recently increasing international interest in traditional uses of medicinal plants on Romanian territory, especially by ethnic minorities [42–48].

Concerning the European ethnopediatry research, there is a relative scarcity of studies. Moreover, the majority of the available European ethnopharmacological field studies only contain isolated references to the medicinal plant use in children diseases, as they are not exclusively focused on traditional ethnopediatry, but rather on adult ethnomedicine. There are such isolated mentions on the traditional indications of medicinal plants in children in various European countries, in cases of digestive diseases [26, 49–59], bedwetting [25], respiratory diseases [55, 60–64], insomnia [52, 63], and growth delay [46, 65]. Nevertheless, more and more scientists try to gather and systematize the available world clinical data on the safety and efficacy of various herbal medicines in children [66–71].

#### Romania—a country with rich biodiversity

Romanian flora has unique diversity, being recently estimated to 3700 species of higher plants [72], including 57 endemic and 171 subendemic species [73]. One of the explanations of this biodiversity lies in the fact that Romania is a biological confluence point, located equally distant from both the North Pole and the Equator and from the Atlantic Ocean and the Ural Mountains. Thus, Romania is the most biogeographically diverse country of the European Union, possessing five of the ten officially recognized geographic regions: alpine, continental, pannonic, pontic, steppic. Moreover, no other European Union country possesses steppe region [72]. Its flora consists of Western and Central European, as well as Mediterranean spontaneous vegetal species, representing more than half of the European flora [30]. More than 700 species were identified as being traditional medicinal plants [74, 75]. A recent evaluation concluded that there are 756 spontaneous medicinal plants in Romania, out of which 126 species are on the Red List, and 122 species are completely forbidden to be collected [76]. Suggestive for the ethnobotanical potential of Romania, is the fact that Romania is on the list of the most important European source regions of medicinal plants collected today from the wild, following behind other Southeastern European countries, such as Bulgaria and Albania [77].

Despite the richness of this biocultural heritage, the studies focused on ethnopediatrics in Romania, as well as in the rest of Europe, are absent. Therefore, we considered that filling this gap represents a priority for scientists. In order to spur the use of Romanian (and European) medicinal plants for the treatment of children diseases, we need first to review the historical use of plants in ethnopediatrics.

The main objective of the present review was to identify, collect, systematize, and prioritize the available bibliographical data related to the medicinal plants traditionally used to treat various pediatric diseases in Romania during the 1860s–1970s.

## Materials and methods

We have obtained ethnobotanical information by manual systematic search in various resources which are not covered in the main electronic databases, such as journal papers, reports, books and PhD works written in Romanian language. All of them, excepting one [11], are available in the Romanian Academy Library (https://biblacad.ro//eng_index.html) and/or in the Romanian National Library (http://www.bibnat.ro/). Depending on the methodology used by the authors of these publications, the resources could be classified into four categories: (A) original publications (based on field study-type methodology) [9–11, 78–84]. Inclusion criterion was that the field work and observations had been performed before 1980, even if some of these studies where published after that date [85, 86] (or the studies included informants who were old enough, at the date of the fieldwork, to report plant use during the target period 1860s–1970s [10, 11, 87]); (B) review publications (based on documentation and synthesis of previous ethnographic or ethnobotanical works) [75]; (C) mixed publications (compilation of original field study and review of literature) [74, 85, 86]; (D) other types of document papers which report use of medicinal plants in children by Romanian people during the 1860s–1970s period [88].

Table 1 contains a brief critical overview of all resources used in this paper. In the following paragraphs, details are provided regarding the seven most representative sources, ordered chronologically according to the date ethnobotanical data were collected or published [whenever the collection date was not available or not applicable (e.g., review work)]:

1. “Botanica Poporană Română” (engl. *Romanian Folk Botany*) by Simion Florea Marian (1847–1907), a Romanian folklorist, ethnographer, hystorian, and naturalist, active member of the Romanian Academy, one of the greatest collectors of information and writers on the Romanian legacy of medicinal plants. Although he was a priest and for the most of his life a schoolteacher, he is the one who layed the foundations of scientific folklore research in Romania. “Botanica Poporană Română” represents a monumental work of therapeutical indications and folklore on medicinal plants in Romanian territory, collected by the author himself from hundreds of informants (whose names and residing villages are mentioned in the book), during 1867–1907. His correspondence attested the fact that he collaborated with specialists from the Botanical Institute in Bucharest to identify (i.e., correctly establish the scientific names of) the plant voucher specimens in his herbarium. Unfortunately, his work was published only recently, one century after his death—this sorrowfully delayed publication was due to the huge efforts of an enthusiastic Romanian ethnographer, Aura Brădăţan [85, 86]. This masterpiece was awarded a distinction by Romanian Science Academy, being considered a treasure of national culture.
2. Istoria naturală medicală a poporului român" (engl. *Natural medical history of Romanian people*) by Nicolae Leon (1862–1931), a Romanian biologist, professor at the Faculty of Medicine, Alexandru Ioan Cuza University, Iasi, published in 1903, in Romanian Academy Annals [84]. He had a very rigurous approach in his fieldwork, in terms of criteria used for plants inclusion in his study. He stated in the Foreword of the publication: “I have not mentioned the vegetal remedies quoted by some authors if I had not the possibility to make sure that the people use them indeed.(…)even if they were counted by Czihak and Szabo as folk medicinal plants(…) Even if they were counted by Czihak and Szabo as folk medicinal plants, the plants that I could not identify because of the lack of voucher specimens are all gathered in Notes, at the end of the Chapter I, and only their use is indicating, without the scientific name.” The publication contains a special chapter entitled “*Numiri vulgare cu cari poporul cunoşte bolele*” (engl. *Folk names by which people know the diseases*), where the author offers the clinical picture of the diseases treated by Romanian folk medicine and also provides the scientific medical terms corresponding to a series of folk terms.
3. “Monografia comunei Răşinariu” (engl. *Monography of Răşinariu village*), by Victor Păcală (1874–1955) [9], awarded a distinction by Romanian Academy in 1916, is considered the best monography of a Romanian village written before the First World War [89], and represents even today a model for how a comprehensive descriptive ethnographic monography should be written.
4. “Contribuţiuni la etnografia medicală a Olteniei” (engl. *Contribution to the medical ethnography of Oltenia*) [82], by Charles Laugier (1875–1930) was granted *Botez Prize* by Romanian Academy in 1927. Charles Laugier was a physician who graduated from Carol Davila Faculty of Medicine in Bucharest in 1898. Latter he became Director and Sanitary Inspector for Oltenia region. During his regular inspections, he collected a lot of ethnographic information, including ethnobotanical and ethnomedical data which is cited even today by specialists. Laugier offers at the end of his publication a list with correspondences between folk terms designating plants/diseases and scientific botanical/medical terms.
5. “Boli, leacuri şi plante de leac cunoscute de ţărănimea română” (engl. *Diseases*, *folk remedies and plants known by Romanian villagers*) published in 1936 by another important figure in Romanian ethnobotany, George Bujorean (1893–1971), botanist and founder of Romanian experimental ecology and geobiology [74]. This publication became a national reference work for its period. Bujorean discovered several new plants with medicinal properties, which were used in Romanian folk medicine, but were not mentioned in Dragendorff’s publication, which was recognized at that time as the world’s ethnopharmacological reference list of medicinal plants [90] (e.g., *Trifolium campestre* L, *Sempervivum marmoreum* Griseb., known at that time by its synonym, *Sempervivum assimile* Schott).
6. “Plantele medicinale si medicina populara la Nişcani” (Engl. *Medicinal plants and folk medicine at Nişcani*) by Alexei A. Arvat (1890-?), a botanist well known in Romanian ethnographic world. He graduated Natural Sciences at Iassy University and published several valuable ethnobotanical works during his life. The one relevant for our present review is a comprehensive field study performed in the Nişcani village, Basarabia, during which he found 140 medicinal species traditionally used against 137 diseases. One of his conclusions is significant for the value of the local ethnobotanical knowledge: the number of medicinal species known to the population of Nişcani was close to the total number of species in that area, his informants claiming that “all the weeds have healing properties, only the people do not know” [80]. He also identified during this study new vernacular names for 41 Romanian medicinal plants. Another interesting conclusion of this work was that medicinal plants were used in folk medicine in Nişcani in a much higher proportion (85%) than other remedies (e.g., incantations- *descântece*, organic or inorganic substances, etc.).
7. “Enciclopedia de Etnobotanică Românească” (engl. *Encyclopedia of Romanian Ethnobotany*) by Valeriu Butura (1910–1989) is a remarkable synthesis work which reveals more than 100 years of traditional Romanian medicine practice [75]. He was a botanist, student of another great Romanian ethnographer Alexandru Borza (1887–1971). Valer Butura started his ethnobotanical studies in 1930–1940 [78, 79, 83]. His encyclopedia containing more than 700 medicinal plants with traditional uses was published in 1979, 10 years after his professor published another reference work *Ethnobotanical dictionary* (containing 2095 species with over 11000 Romanian names of plants) in 1968 [91].

**Table 1:** Resources used for the historical review on medicinal plants used in Romanian ethnopediatry in 1860s–1970s

Title	Author	Year of publication	Sources (and methodology)	Trust level in terms of plant (criterion 1+ criterion2) and disease identification	Reference
1) “Botanica Poporană Română” (engl. *Romanian Folk Botany*)	Simion Florea Marian	2008, 2010 (post-mortem publication; data collected during 1867-1907)	Fieldwork (observational method, collection of plant voucher specimens); literature (review)	5 (2+3) (plants) 3 (diseases)	[85, 86]
2) “Istoria naturală medicală a poporului român” (engl. *Natural medical history of Romanian people*)	Nicolae Leon	1903	Fieldwork (observational method, identification of plants based on collection of voucher specimens)	6 (3+3) (plants) 4 (diseases)	[84]
3) “Monografia comunei Răşinariu” (engl. *Monography of Răşinariu village*)	Victor Păcală	1915	Fieldwork (ethnographic method- participant observation, key informant interviewing; complete inventory of local flora)	4 (1+3) (plants) 2 (diseases)	[9]
4) “Contribuţiuni la etnografia medicală a Olteniei” (engl. *Contribution to the medical ethnography of Oltenia*)	Charles Laugier	1925	Fieldwork (observational method)	4 (1+3) (plants) 4 (diseases)	[82]
5) “Boli, leacuri şi plante de leac cunoscute de ţărănimea română” (engl. *Diseases, folk remedies and plants known by Romanian villagers*)	George Bujorean	1936	Fieldwork (observational method); literature (review)	6 (3+3) (plants) 4 (diseases)	[74]
6) “Plantele medicinale şi medicina populară la Niscani” (Engl. *Medicinal plants and folk medicine at Niscani*)	Alexei A. Arvat (1890-?),	1937	Fieldwork (observational method, key informant interviewing, inventory of ethnobotanical and ethnographic data, collection of plant voucher specimens)	6(3+3) (plants) 4 (diseases)	[80]
7) “Enciclopedia de Etnobotanică Românească” (engl. *Encyclopedia of Romanian Ethnobotany*)	Valer Butura	1979	Literature (review)	6 (3+3) (plants) 4 (diseases)	[75]
8) “Plante cunoscute şi întrebuinţate de românii din Ardeal. Note etnobotanice” (engl. *Plants known and used by Romanians in Ardeal. Ethnobotanical notes*)	Valer Butura	1935	Fieldwork (observational method)	6 (3+3) (plants) 3 (diseases)	[83]
9) “Plante cunoscute şi întrebuinţate de românii din Transilvania” (engl. *Plants known and used by Romanians in Transilvania*)	Valer Butura	1936	Fieldwork (observational method)	6 (3+3) (plants) 4 (diseases)	[79]
10) “Plante cunoscute şi întrebuinţate de locuitorii câtorva sate româneşti (Ethnobotanische mitteilungen aus Rumanien)” (engl. *Plants known and used by inhabitans of few Romanian villages*)	Valer Butura	1938	Fieldwork (observational method)	6(3+3) (plants) 4 (diseases)	[78]
8) “Florile bune de leac” (engl. *Flowers good for healing*)	George Ulieru	1929	Medical literature essay	2 (1+1) (plants) 4 (diseases)	[88]
9) “Noutati etnobotanice româneşti” (engl. *Romanian ethnobotanical novelties*)	Alexandru Borza	1936	Fieldwork (observational method, collection of plant voucher specimens)	6 (3+3) (plants) 3 (diseases)	[81]
10) “Studii de etnobotanică în comuna Poiana Cristei, jud. Vrancea” (engl. *Ethnobotanical studies in Poiana Cristei village, Vrancea county*)	Ana Condrea	1991	Fieldwork (observational method, old informant interviewing)	6 (3+3) (plants) 4 (diseases)	[87]
11) “Valea Sebeşului. Monografie Etnofolclorică, vol.II. Folclor” (engl. *Sebeş valley. Ethnofolklorical monography, vol. II, Folklor*)	Gheorghe Pavelescu	2004 (data collected in 1934-1939, and enriched in 1971)	Fieldwork (observational method, informant interviewing)	5 (3+2) (plants) 2 (diseases)	[10]
12) “Medicina populară din Basarabia de la sfârșitul secolului al XIX-lea – începutul secolului al XX-lea. Aspecte istorico-etnografice” (engl. *Folk medicine from Basarabia at the end of the 19th - beginning of the 20th centuries. Historical-ethnographical aspects*)	Natalia Gradinaru	2015 (data collected during 2003-2014; average age of informants 71.5 years)	Fieldwork (observational method, questionnaires, simulation- to reveal “professional secrets”, case study); literature (review of field studies available in the Archives of the Institute of the Cultural Heritage, Chişinău )	6(3+3) (plants) 4 (diseases)	[11]

We estimated for each source used the trust level in terms of plant identification, using two graded criteria, as follows:

Criterion 1 (author’s background): 3—author was botanist, biologist, or anthopologist; 2—the author was not a botanist, biologist, or anthropologist, but collaborated with botanists for the identification of plants; 1—author was a physician or self-educated in terms of botanical and anthropological studies; 0—author was none of the previous.

Criterion 2 (plant identification): 3—if the author was able to differentiate between medicinal plants with claimed clear botanical identity and plants with unresolved botanical identity, and *all* the plants with claimed clear botanical identity had Latin names which were either accepted names or synonyms of the accepted names in The Euro+Med PlantBase (http://ww2.bgbm.org/EuroPlusMed) [92] and/or The Plant List (www.theplantlist.org) [93]; 2—if the Latin names of the *majority* of medicinal plants claimed to have a clear botanical identity were either accepted names or synonyms of the accepted names in The Euro+Med PlantBase (http://ww2.bgbm.org/EuroPlusMed) [92] and/or The Plant List (www.theplantlist.org )[93]; 1—if the Latin names of the medicinal plants were available for a small percentage of taxa, or a significant number of plant had unresolved names in The Euro+Med PlantBase (http://ww2.bgbm.org/EuroPlusMed) [92] and/or The Plant List (www.theplantlist.org) [93], or Latin names were not available (and plant identification was based on the vernacular name).

The sum of grades for the two criteria represented the trust level in terms of plant identification. The highest possible grade was 6 (3 + 3), and the lowest possible grade was 1 (0 + 1).

Whatever the trust level of the source, we did not include plants with unclear botanical identity in our work.

We estimated for each source also the trust level in terms of disease identification, as follows: 4—in the source, the diseases are identified by their scientific names or by both their folk and scientific names; 3—some diseases are identified by their scientific names, others only by their folk names; 2—diseases are identified only by their folk names, whose modern medical equivalents could be determined nonetheless by means of dictionaries or other resources [74, 84, 94]; 1—diseases are designated only by folk terms whose significance in modern medical terms could not be established with the help of dictionaries or other resources. The higher the grade, the higher the trust level. The highest possible grade was 4, and the lowest possible grade was 1.

A medicinal plant was included in our database if its traditional use was mentioned by at least one author.

Despite the fact that the majority of the dietary plants may be used for children, those species where pediatric indications were implicit (due to their dietary value) were excluded from our study, while only the species with explicit pediatric indications (mentioned in the ethnographic text as such) were included. For instance, plants like *Urtica dioica*, *Malus domesticus*, *Vitis vinifera*, *Persica vulgaris*, *Petroselinum sativum*, *Raphanus sativum*, etc. were excluded, despite their ethnopediatric potential. Beside the objective restrictions imposed by the limited availability of written resources, this approach was adopted in order to (1) reduce to zero/nullify the risk of selecting a wrong plant, (2) avoid overloading the paper with too many common plants with universal dietary value, and (3) identify local non-nutritional medicinal plants with pediatric indications, which might be less known to the scientific community.

Regarding the name of the plants—in our sources a perimated Latin term was sometimes used for species identification. In all these cases, the perimated Latin term was changed to the presently accepted one (e.g., *Galium odoratum* (L.) Scop. instead of *Asperula odorata* L.). More often than not, a given species had several vernacular names—in such cases, all the various vernacular names encountered in our sources were gathered as a unique entry under the accepted Latin name (e.g., “sânziene de pădure,” “vinariţă,” and “mama pădurii” are put together under the accepted latin name *Galium odoratum* (L.) Scop.).

Data analysis and extraction were performed by medical professionals involved in clinical and scientific research at academic level. All data were cross examined by a second author.

Concerning the indigenous classification of diseases in Romanian traditional medicine, to the best of our knowledge, no systematic classification is provided by the available historical resources. In order to avoid suppressing the cultural traits, we have adapted the International Classification of Primary Care (ICPC) [95] to our set of data. ICPC was reported to be a closer approximation to ethnomedical reality and emic perspective, than other modern classifications such as International Statistical Classification of Diseases and Related Health Problems (ICD) or the Economic Botany Data Collection Standard (EBDCS) [96].

Preliminary data concerning traditional pediatric indications and the parts used were organized in a tabular form. Afterwards, we performed a second systematic search in PubMed for all the medicinal plants recorded for ethnopediatric use which were included in our database, in order to identify whether their therapeutic potential was evaluated or not in clinical pediatric studies.

### Data analysis

We divided the collected bibliographic material into use categories such as various types of diseases, based on the International Classification of Primary Care (ICPC) (WHO | International Classification of Primary Care, Second edition (ICPC-2), 2012).

In order to compare the utility of medicinal plants in Romanian ethnopediatry, we proposed one index: Ethnopediatric Relative Therapeutic Versatility (ETRV).

In our analysis, all ethnopedriatic uses included under the umbrella of a certain targeted body system were counted as one. For each medicinal plant, we summed up all the targeted body systems and obtained a value designated as BS. Phylogenetically closely related medicinal plants (the single case of *Populus* spp.), which had similar ethnomedical uses, were counted together, as a single phytotherapic entity. The formula used for the calculation of ERTV expressed as percentage was:

$$ \mathrm{ERTV}\%=\frac{ BS i}{BS\max}\times 100 $$

where BS_i_—number of body systems targeted by the plant i; BS_max_—maximum number of body systems targeted by a single plant obtained in our bibliographic study (which is seven). For example, *Gratiola officinalis* is traditionally used to treat six body systems, one less than 7, corresponding to *Dryopteris filix-mas* (*L*.) *Schott*, the most versatile of all species. Therefore, it has ERTV of 85.71% (6:7 ×100).

Altogether, more than 30 local publications were used for this historical review to collect information about medicinal plants used in Romania (1860s–1970s), but only in 15 publications we have found relevant data for ethnopediatrics. Since some of the publications rely on identical sources (e.g., George Bujorean [74] is cited by other ethnographers, such Valer Butura [75]), we considered that the number of citations did not express the relative importance of the species in Romanian ethnopediatrics.

## Results

We have identified in the available literature several old Romanian terms comprising children-specific diseases (*acrum*—newborn aphthous stomatitis, *babiţi*—digestive troubles in children caused by tooth eruption, *boala cânească/socote/sohote/zilizit—*athrepsia, severe nutritional disturbance in small children, *boala copiilor/răul copiilor/răutatea copiilor/ceas rău/samca/sanca—*convulsions, epilepsy, *coriu/coriu adevărat/bubatu al mic—*measles, *coriu negru—*scarlet fever, *focușor—*red papular eruptions on face or chest in children, *lamoste—*child dysentery, *mătrice—*cramps in newborns due to abdominal gas accumulation, *muma pădurii*—nightmares/insomnia/weeping during night in small children, *oase moi* (“soft bones”)/oase strâmbe (“curved bones”)—rickets, *opăreală—*diaper/napkin dermatitis, *rahnă—*cold, *pleasnă/plesne—*irritation of lingual and palatal mucosa or aphtous stomatitis, especially in small children, *rodimcic*—nervous spasms, convulsions, cramps in newborns, *strâns*—diarrhea of children, *suldumaș or surdumaș—*small red papular eruption on the newborn scalp, *tuse măgărească—*whooping cough) or disorders common in both adults and children (*apucătură-* colic*, arâne/fudulie-* scabies*, boli lipicioase-* contagious diseases*, bubă-* abscess/pustule/purulent subcutaneous collection, *bube dulci/rofii/rohii—*impetigo, *cufureală/treapăd*—diarrhea, *gîlci*—tonsillitis, *izdat/surdumaci—*abdominal pains, *mărgăritărel—*stomatitis, *scrofuri—*tuberculosis of the lymph nodes, *trecătură*—gastro-enteritis) [74, 75, 85, 94, 97]. Some of them are still used in the modern language (e.g., *tuse măgărească*, *bube dulci*, *cufureală*, *gîlci*).

After adaptation of the International Classification of Primary Care (ICPC) [95] to our set of data, we had 12 categories of body system-related pediatric diseases (Table 1).

A total of 153 medicinal plants belonging to 52 families were identified as having ethnopediatric relevance—they are presented in the alphabetical order of their Latin name in Table 3, which also includes the medical indications, targeted body systems, parts used, way of administration (where available), and ERTV. Half of the species (49.67%) used in Romanian traditional medicine to treat children diseases belong to seven families: *Asteraceae* (21), *Lamiaceae* (15), *Apiaceae* (12), *Rosaceae* (ten), *Fabaceae* (seven), *Ranunculaceae* (six), *Brassicaceae* (five).

The plant indications, targeted body systems, parts used, way of administration (where available), ERTV, and available scientific evidence are provided in Table 2.

**Table 2:** Classification of ethnopediatric indications in the present study, adapted after the International Classification of Primary Care (ICPC)

Category	Examples of diseases
General and unspecified	Asthenia, Chicken pox/Varicella, Scarlet fever, Colic, Cramps, Fever, Measles/Rubeola, German measles/Rubella, Spasms, Tuberculosis, Weakness, Physical debility
Blood, blood forming organs, lymphatics and spleen	Anemia, Enlarged lymph nodes, Scrophulosis
Cardiovascular	Tachycardia
Digestive	Abdominal cramps, Abdominal pain, Acute digestive infections, Colitis, Constipation, Dental abscesses, Dental cavities, Dental eruption, Diarrhea, Digestive cramps, Dysentery, Enteritis, Flatulence, Intestinal cramps, Intestinal parasites, Intestinal worms, Stomatitis, Tape worms, Teeth ache/pain, Ulcerative stomatitis, Vomiting
Ear	Ear discharge, Ear pain
Musculoskeletal	Bone diseases, Bone deformities, Disability/Weakness of the extremities Rickets, Trauma
Neurological	Convulsions, Epilepsy, Paralysis
Psychological	Agitation, Anxiety/Fright, Enuresis, Irritability, Sleep disturbances/Insomnia/ Nightmares/weeping during sleep, Psychosis
Respiratory	Acute respiratory diseases, Asthma, Bronchitis, Phlegm in the throat, Cold, Cough, Cough with sputa and puss, Whooping cough, Respiratory infections, Tonsillitis
Skin	Acne, Burn wounds/Burns, Dermatitis, Diaper (napkin) dermatitis, Eczema, Hair complaint, Skin inflammation, Skin infections, Impetigo, Scabies, Skin lesions, Subcutaneous tumors, Verruca, Wounds
Endocrine, metabolic and nutritional	Anorexia/Loss of appetite, Athrepsia, Cashexia, Growth dysfunctions/Growth delay, Underweight, Nutritional dysbalances
Urology	Anuria, Oliguria

The distribution of the plants with respect to their medical indications is as follows: blood, lymph and spleen diseases (five species), cardiovascular (one), digestive (53), ear (two), endocrine, metabolic, and nutritional (28), general (45), musculoskeletal (11), neurological (22), psychological (26), respiratory (14), skin (44), urological diseases/conditions (one).

**Fig. 1:**
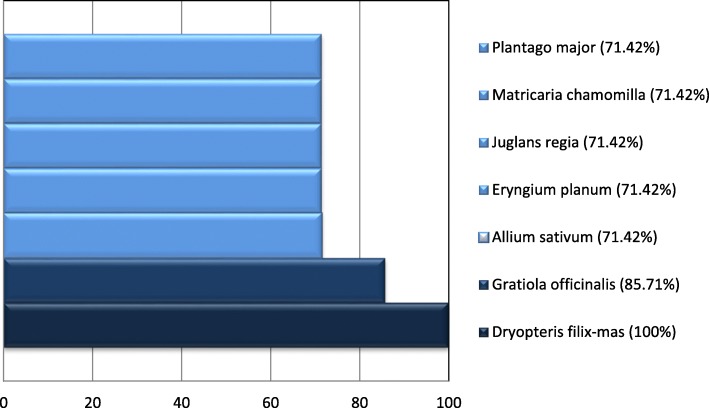
Top 7 of the most versatile plants in Romanian ethnopediatry (Ethnopediatric Relative Therapeutic Versatility- ERTV- expressed as percentage)

The top of the seven most versatile plants in Romanian ethnopediatry is represented in Fig. 1.

Only 15 medicinal plants were scientifically evaluated in pediatric clinical studies, or at least in human clinical studies with mixed groups of subjects (adults and children). For six plants, we found some positive evidence for few indications mentioned in Romanian ethnopediatrics; for ten medicinal plants, some positive evidence for certain indications not mentioned in Romanian ethnopediatrics, and for one plant some negative evidence.

## Discussion

Ethnopediatrics in Europe, as well as in Romania, represents a neglected field of research. Many potential reasons could explain this fact. Regarding clinical trials (not only with herbal medicines, but also with synthetic drugs) in this vulnerable population, we should take into account, first of all, that The European Pediatric Regulation was adopted only recently, in 2006, and entered into force in 2007 [120]. Moreover, scientists face multiple challenges related to small subjects’ population, economical burdens, pharmacological issues (extra-toxicity test required, higher risks of unpredictable severe adverse reactions in children than in adults), ethical concerns, necessity of additional parental consent, multiple age groups with specific needs and dramatic differences between them, difficulties in communication with children, high percentage (60%) of children who do not assent to take part in research studies, high rate of withdrawal, etc. [121, 122]. Owing to all these constraints, it was suggested to use all available knowledge (e.g., clinical pharmacology data generated by using many therapeutic agents with no approved dosing labels and guidance), as well as modeling and simulation approaches in drug development [123]. We have proposed here a complementary solution to fill the actual gap in the pediatric research: to use all available, but neglected ethnopediatric knowledge, starting with systematic analysis of historical resources, and following with field studies. The present paper follows this direction, focusing on the review of medicinal plants used in Romanian ethnopediatrics in the 1860s–1970s.

More than 700 common species of medicinal plants are listed in various resources as being used in Romanian traditional medicine [74, 75, 124]. According to the present study, around one-fifth of them are highlighted as having ethnopediatric indications. We have also noticed that a great part of the indications of phytoremedies in children are for acute diseases: from a total of approximately 80 different types of illnesses, about two-thirds were acute (e.g., acute respiratory diseases, acute digestive disorders with vomiting and diarrhea, intestinal colic and other types of pains, spasms, wounds, chicken pox, scarlet fever, dental eruption, etc.) (see Table 1). This fact is expected since children often have acute, short-term illnesses [125]. Also, some of the most common adult diseases that are chronic (e.g., cardiovascular, central nervous system and oncological diseases) are less frequent in children [126].

Some of these plants have been cultivated and have been part of everyday Romanian diet for many centuries: aromatic plants (e.g., *Foeniculum vulgare*, *Mentha piperita*, *Levisticum officinale*), fruits and seeds (e.g., *Pyrus communis*, *Rosa canina*, *Juglans regia*, *Cucurbita pepo*), vegetables (e.g., *Zea mays*, *Phaseolus vulgaris*, *Vicia faba*, etc.), various recipes being preserved during the centuries, especially in the rural communities [30, 127]. Some particular Romanian plant-based dishes, which are also consumed by children, are *pumpkin pie* (from Muntenia area), *bean soup* (from Transylvania), *home*-*made noodles* (“*iofca*”) prepared with cabbage, nuts, poppy (from Banat) [127]. Interestingly, the experts in food sciences suggested that the traditional cooking methods and habits seem to selectively preserve the specific benefic activities of different phytochemicals [128, 129]; therefore, we suggest that studying the traditional recipes may inspire new extraction methods of bioactive constituents.

Concerning the fact that only some of the plants were specifically marked for use in children in the studied book and papers, a question arises: what is the reason or reasons behind this specification? We can only hypothesize. Potential explanations are the following: these plants might have been more frequently used or considered to be more efficient in their therapeutic activity by the informants or ethnographers, the informant might had a special direct experience with that plant, a certain plant or plant use might had a special value for a local community or for a family of healers, transmitted information might have been uncomplete due to weak memory of the informant. It was also suggested that the traditional ethnopharmacological knowledge is unevenly distributed (e.g., women, as managers of household health and mothers, might know more about ethnopediatric remedies than men; differential acces to the landscapes, such as forests, and therefore to certain medicinal plants; increased amount of knowledge with age of informant, etc.) [130–133].

### Ways of medicinal plant administration in ethnopediatry in Romania

In Romanian ethnopediatry, medicinal plants are prepared in various ways, which are intended for internal use (infusions, decoctions, syrups), external use (baths, ointments, cataplasms, fumigations, inhalations), or both (infusions, decoctions). Alcoholic preparations are in general not recommended in Romanian (ethno)pediatry [85]. The reason is obvious, since alcohol is known to be toxic when administered to children [134, 135]. WHO proposed the limitation in the ethanol content of pediatric products to less than 0.5% [136]. We noticed that the most frequently cited way of administration for small children in Romanian ethnopediatry was by bath, not only for skin diseases, but also for internal diseases, such as digestive or neurological ones, due to the fact that the quantities of phytochemicals absorbed through the skin, albeit small, are sufficient to be active in young children, particularly because phytochemicals can reach the bloodstream easier through the skin in small children than by internal administration in adults [137–139]*.*

### Use of toxic plants in Romanian ethnopediatry

In our bibliographic study, we have noticed that in Romanian ethnopediatry some of the toxic alkaloid-rich species (*Aconitum napellus*, *Dryopteris filix-mas*, and *Tanacetum vulgare*) have been traditionally prescribed under rigorous dosing, with the specification of increased toxicity [75, 86, 124], some of them even for internal use (e.g., *Acontium napellus* for epilepsy, *Dryopteris filix*-*mas* for scrophulosis)! We have noticed in some of these cases that the traditional recipe is usually a polyherbal multimineral formula, with a complicated way of preparation. The rationale behind this may be the reduction of plant/mineral toxicity, similarly to the herbo-mineral ayurvedic preparations called *rasa oushadies* [140]. We give one such example from Romanian ethnomedicine. In Bucovina, the peasants used to prepare a decoction of wolfsbane (*Aconitus napellus L.*) and silver thistle (*Carlina acaulis L.*), over which they added *sineala* (*syn.* ultramarine), a blue mineral dyestuff used in the past to bleach the laundry, obtained by melting a mixture of kaolin, sodium carbonate, wood ash, and sulfur*.* This complex liquid preparation was administered to the child suffering from epilepsy (Rom. *raul copiilor*), both internally (a very small amount) and externally (as a whole body washing) [86].

### Comparison of relative therapeutic versatility of top medicinal plants in Romanian ethnopediatry

Concerning the ERTV index, we discovered several interesting facts. Surprisingly, the versatility hierarchy is topped by two less commonly used medicinal plants with toxic potential: fern (*Dryopteris filix-mas*) with ERTV 100% and gratiole (*Gratiola officinalis*) with ERTV 85.71%. Not unexpectedly, next to these two species were four common medicinal species, used in many parts of the globe in ethnopediatry [garlic (*Allium sativum*), nut tree (*Juglans regia*), chamomile (*Matricaria chamomilla*), and broadleaf plantain (*Plantago major*)], and another one less known for its ethnopediatric use, blue eryngo (*Eryngium planum*), all having equal scores (ERTV 71.42%). *Dryopteris filix*-*mas*’ biological activities are not yet scientifically studied, except for its antiinflammatory potential (one animal study) [141]. *Gratiola officinalis*’ therapeutic activity is more evidence based; its anti-inflammatory (correlated with its ethnopediatric use in eczema, see Table 3) and sedative (correlated with its ethnopediatric indication in fright during sleep, see Table 3) potential being partially supported by animal or in vitro studies [142, 143]. Nevertheless, its ethnopediatric use in physical debility, epilepsy, paralysis, and tachycardia has no scientific support yet.

**Table 3:** Medicinal plants used in ethnopediatrics in Romania (1860s–1970s)

No.	Species	Family	English name	Romanian name	Origin	Traditional indications and targeted body systems	Parts used	ERTV (%)	References for traditional indications	Scientific evidence in pediatric clinical studies
1.	*Acer campestre* L.	Sapindaceae	Field maple	Jugastru	Native	1. General: Weakness (for general strengthening; ext-bath, int-sap)	Bark, Sap	14.28	[75, 86]	n.y.s.
2.	*Aconitum napellus* L.	Ranunculaceae	Wolfsbane	Omag, toaie	Native	1. Neurological: Epilepsy (int., ext-bath) 2. Skin (ext): Wounds	Leaves, Root	28.57	[75, 86]	n.y.s
3.	*Allium ascalonicum* L.	Amaryllidaceae	Shallot	Haşmă, hajme, hagima	Non-native	1. Skin: Eczema, Impetigo (ext), scalp red eruptions of newborn	Bulb, leaves	14.28	[75, 84, 86]	n.y.s.
4.	*Allium cepa* L*.*	Amaryllidaceae	Onion	Ceapă	Non-native	1. Respiratory: Asthma, Bronchitis, Cough; 2. Digestive: Stomach pain, intestinal colic (ext + int), Tape worms, Cramps in newborns due to accumulation of gas (int- 1 drop juice of onion), Ulcerative stomatitis, Dental eruption; 3. Ear: pain; 4. Skin: newborn scalp eruptions (ext)	Bulb, leaves	57.14	[9, 75, 84–86]	p.e. Skin: alopecia areata- effective topical therapy (Note. mixed group of adult and child subjects) [98]
5.	*Allium sativum* L.	Amaryllidaceae	Garlic	Usturoi	Non-native	1. Digestive: Intestinal worms, Diarrhea, Gastro-enteritis, Colic 2. Respiratory: Cough with sputa and puss 3. Skin (ext): Verruca, Skin infections, Impetigo 4. Psychological: nightmares 5. Neurological: : epilepsy (ext-polyherbal ointment, in combination with lovage, elecampane, and other plants with unresolved botanical identity)	Bulb, leaves	71.42	[74, 75, 82, 84, 86]	p.e. antibacterial n.e. antifungal- as polyherbal mouth rinse [99]; p.e. antiverruca [100]; p.e. respiratory diseases (improved oxygenation and dyspnea in children with hepatopulmonary syndrome) [101]; n.e. topical non-effective therapy in alopecia areat a[102]
6.	*Althaea officinalis* L*.*	Malvaceae	Marsh mallow	Nalbă mare	Native	1. Digestive: Diarrhea 2. Skin: diaper (napkin) dermatitis	Flowers, leaves, roots	28.57	[75, 88]	n.y.s
7.	*Amygdalus communis* L.	Rosaceae	Almond tree	Migdal	Non-native	1. Ear: Ear pain, Ear discharge	Buds, seeds, flowers, bark	14.28	[86]	*p.e.-↓ symptoms in attention-deficit/hyperactivity disorder [103]
8.	*Anemone pulsatilla* L.	Ranunculaceae	Pasque flower	Dediţel, sisinel	Native	1. Psychological: Insomnia (ext-fumigations)	Flowers	14.28	[75]	n.y.s.
9.	*Anethum graveolens* L.	Apiaceae	Dill	Mărar	Native	1. Digestive: Abdominal cramps, Colic, Intestinal worms (roundworms)	Aerial parts, Seeds	14.28	[75, 84, 86]	p.e. antiparasitic ( ↓ incidence of Giardia lamblia after 5 days of treatment) [104]
10.	*Anthyllis vulneraria* L*.*	Fabaceae	Woundwort	Vătămătoare	Native	1. Skin: Eczema, Wounds (ext); 2. Psychological: Fear, Fright (int)	Flowering tips	28.57	[75, 83]	n.y.s.
11.	*Aquilegia vulgaris* L.	Ranunculaceae	Columbine	Căldăruşă	Native	1. Respiratory: Whooping cough (ext)	Aerial parts	14.28	[75]	n.y.s.
12.	*Arctium lappa* L.	Asteraceae	Greater burdock	Brusture	Native	1. Skin: Wounds; infections; Eczema; 2. Digestive: cramps (ext)	Leaves	14.28	[75, 86]	n.y.s.
13.	*Arnica montana* L*.*	Asteraceae	Mountain arnica	Arnică	Native	1. Musculoskeletal: Trauma (ext); 2. Psychological: Anxiety/ fright (only the plant collected on the Cross Day) (ext- fumigations)	Leaves	28.57	[75, 85]	n.y.s.
14.	*Artemisia abrotanum* L.	Asteraceae	Sagebrush	Lemnul domnului, lemnuş	Native	1. General : weakness (ext-weekly bath, flowering tips) 2. Digestive: Stomatitis (ext- leaves in honey, chewing twigs), colic (int- milk decoction of bark) 3. Musculoskeletal: riketts (ext-bath weekly, flowering tips)	Leaves, Flowering tips, Bark	42.85	[75, 84, 86]	n.y.s.
15.	*Artemisia absinthium* L.	Asteraceae	Wormwood	Pelin	Native	1. Nutritional: Athrepsia (ext- crushed fresh leaves) 2. Neurological: Epilepsy (ext-bath)	Leaves	28.57	[74, 75, 84]	n.y.s.
16.	*Aruncus dioicus* (Walter) Fernald	Rosaceae	Goat's beard, bride's feathers	Coada priculicilor, barba popii	Native	1. Neurological: Epilepsy (ext- bath, fumigation) 2. Psychological: Insomnia (ext- bath, fumigation)	Aerial parts	28.57	[85]	n.y.s.
17.	*Astragalus glycyphyllos* L.	Fabaceae	Wild liquorice	Unghia găii, unghia căţelei, iarba limbricilor	Native	1. Skin (ext): Eczema, Diaper (napkin) dermatitis (washing with decoction in milk), Necrotic wounds (Rom. “*colţ de lup*”- cutaneous tuberculosis or cutaneous lesions in syphilis), Infections, Panaris	Aerial parts	14.28	[75, 78, 80, 84]	n.y.s.
18.	*Ballota nigra* L.	Lamiaceae	Black horehound	Urzică moartă, cătuşe	Non-native	1. General: for suffering children (ext-bath)	Aerial parts	14.28	[80]	n.y.s.
19	*Berteroa incana* (L.) DC.	Brassicaceae	Hoary alyssum	Ciucuşoară, păsatul vraghiei	Native	1. Skin: infections (ext ), eczema (ext-bath)	Aerial parts	14.28	[75, 80]	n.y.s.
20.	*Betula pendula* Roth	Betulaceae	Silver birch	Mesteacăn	Native	1. Nutritional: Athrepsia, Nutritional dysbalances, growth delay (ext- sap or bath with decoction of bark from a young tree)	Sap of the young tree, bark of a young tree	14.28	[9, 74]	n.y.s.
21.	*Betula pubescens* Ehrh.	Betulaceae	White birch	Mesteacăn alb	Native	1. Nutritional: Athrepsia, Nutritional dysbalances, Growth delay (ext-bath)	Sap of the young tree	14.28	[74]	n.y.s.
22.	*Bidens tripartitus* L.	Asteraceae	Three-lobe beggarticks	Dentiţă, cîrligei, turiţă	Native	1. General: Weakness (for general tonification/strengthening)	Stems, leaves, flowers	42.85	[75]	n.y.s.
23.	*Brassica oleracea* L.	Brassicaceae	Cabbage	Varză	Non-native	1. Skin: Wounds, Impetigo (ash of burnt cob) 2. General: Measles	Leaves	28.57	[84, 86]	n.y.s.
24.	*Butomus umbellatus* L.	Butomaceae	Flowering rush	Crin de baltă, roşăţea	Native	1. Neurological: Convulsions, Epilepsy (ext-bath)	Aerial parts	14.28	[75]	n.y.s.
25.	*Calendula officinalis* L.	Asteraceae	Common marigold	Filimină, Gălbinele	Native	1. Psychological: Insomnia (ext-bath)	Flowers	14.28	[75]	p.e.* ↓ severity of diaper dermatitis [105–107]; p.e.* ↓ clinical signs of chronic blepharitis and dry eye syndrome (Note. Mixed group of subjects-adults and children [108];
26.	*Cannabis sativa* L.	Cannabaceae	Hemp	Cânepă	Native	1. Skin (ext): Infections, Impetigo	Seeds	14.28	[74]	p.e.*benefits in epilepsy [109]; p.e.*motor disorders (improved spasticity and dystonia, sleep disturbances, pain severity) [110]
27.	*Carduus nutans* L.	Asteraceae	Musk thirstle	Scăiete, Ciulin	Native	1. General: Weakness (for general tonification/strengthening)	Aerial parts	14.28	[75]	n.y.s.
28.	*Carlina acaulis* L.	Asteraceae	Silver thirstle	Ciurul zânelor, sita ielelor	Native	1. Neurological: Epilepsy (ext-bath, int); 2. Psychological: Fright (ext-fumigations)	Flowers	28.57	[84, 86]	n.y.s.
29.	*Carum carvi* L.	Apiaceae	Caraway	Chimen	Native	1. Digestive: Cramps, Colic, Diarrhea, Flatulence	Seeds	14.28	[74, 84]	n.y.s.
30.	*Centaurium erythraea* Rafn	Gentianaceae	European centaury	Ţintaură	Native	1. General: Weakness 2. Endocrine/Metabolic and Nutritional: Anorexia (int-syrup) 3. Neurological: Epilepsy (ext-bath)	Stems, leaves, flowers	42.85	[75, 84]	n.y.s.
31.	*Ceratocephalus falcatus* (L.) Pers.#	Ranunculaceae	-	Ploşnicar	Native	1. Skin (ext): Infections	Aerial parts	14.28	[75]	n.y.s.
32.	*Chaerophyllum aromaticum* L.	Apiaceae	-	Antonică	Native	1. Digestive: Tooth pain 2. Neurological: Headache 3. Psychological: Psychosis	Leaves	42.85	[85]	n.y.s.
33.	*Chelidonium majus* L.	Papaveraceae	Celandine	Rostopască	Native	1. General (root, ext- bath for general tonification/strengthening)	Root	14.28	[75]	p.e.* chronic tonsillitis (improved cellular and humoral immunity, nonspecific resistance, reduced the number of recurrences) [111]
34.	*Cichorium intybus* L.	Asteraceae	Commom chicory	Cicoare	Native	1. Neurological: Epilepsy (ext- bath)	Leaves, roots, rhizome	14.28	[85, 86]	p.e.* acute gastroenteritis-related diarrhea (reduced duration of acute diarrhea) [112]
35.	*Cirsium arvense* (L.) Scop.	Asteraceae	Creeping thirstle	Pălămidă, pălămidă seacă	Native	1. Nutritional: Athrepsia (ext-bath)	Aerial parts, roots	14.28	[74]	n.y.s.
36.	*Cirsium oleraceum* (L.) Scop.	Asteraceae	Cabbage thirstle	Crăpuşnic	Native	1. General: for general strengthening/tonification (ext-bath)	Roots, stems	14.28	[85]	n.y.s.
37.	*Clematis vitalba* L.	Ranunculaceae	Old man’s beard	Curpen	Native	1. General: Weakness (ext)	Aerial parts	14.28	[75, 84, 85]	n.y.s.
38.	*Cochlearia officinalis* L*.*	Brassicaceae	Commom scurvygrass	Lingurea	Native	1. General: Tuberculosis 2. Digestive: Indigestion/Dyspepsia 3. Endocrine, metabolic, nutritional: Lack of appetite	Leaves	42.85	[75]	n.y.s.
39.	*Conium maculatum* L.	Apiaceae	Hemlock	Cucută	Native	1. Skin (ext): Dermatosis 2. Neurological: Paralysis (ext- bath)	Leaves	28.57	[75]	n.y.s.
40.	*Consolida regalis* Gray	Ranunculaceae	Forking larkspur	Somnoroasă, creasta cucului	Native	1. Psychological: insomnia (int- decoction)	Flowers	14.28	[80]	n.y.s.
41.	*Cornus mas* L*.*	Cornaceae	European cornel	Corn	Native	1. Digestive: Diarrhea 2. Psychological: Agitation with screaming 3. General: Typhoid fever 4. Nutritional: Underweight	Fruits	57.14	[75, 84–86]	p.e.* improvement of lipid profile and vascular inflammation [113]
42.	*Corylus avellana* L.	Betulaceae	Common hazel	Alun	Native	1. General: Weakness (ext-bath for general tonification/strenghtening)	Leaves, buds, fruits, male flowers	14.28	[74, 75]	n.y.s.
43.	*Cucurbita pepo* L.	Cucurbitaceae	Pumpkin	Bostan	Non-native	1. Endocrine, metabolic, nutritional: Underweight	Pulp	28.57	[75]	n.y.s.
44.	*Daucus carota* L.	Apiaceae	Carrot	Morcov	Non-native	1. Musculoskeletal: Rickets 2. Digestive: Stomatitis 3. Skin: Wounds, Impetigo (ext)	Roots, leaves, seeds	42.85	[75, 84, 86]	p.e.*gastroenteritis (stools returning to normal consistency and frequency in 6 days) [114]
45.	*Dryopteris filix-mas* (L.) Schott	Dryopteridaceae	Male fern	Ferigă	Native	1. Digestive: Intestinal worms 2. Skin (ext): Wounds 3. Neurological: Epilepsy; 4. Endocrine, metabolic and nutritional (ext-bath): Delayed growth 5. Psychological (ext): insomnia 6. Musculoskeletal (ext): Rickets, Bone diseases, Bone deformities 7. Blood and lymph nodes: Scrophulosis (ext-cataplasm + int- milk decoction)	Rhizome, leaves	100	[75, 84]	n.y.s.
46.	*Equisetum arvense* L.	Equisetaceae	Common horsetail	Coada calului	Native	1. Digestive: Diarrhea	Sterile aerial parts	28.57	[75]	n.y.s.
47.	*Equisetum fluviatile* L.	Equisetaceae	Water horsetail	Pipirig	Native	1. Musculoskeletal: walking difficulties (bath for bone strengthening, in combination with *Jacobaea erratica* (Bertol.) Fourr.))	Not specified (probably aerial parts)	14.28	[75, 79]	n.y.s.
48.	*Eryngium planum* L.	Apiaceae	Blue eryngo	Scai vânăt, spinul albastru	Native	1. Endocrine, metabolic, nutritional: Underweight 2. Skin (ext): Scalp fungal infections, Impetigo, Eczema 3. General: Weakness (ext-bath for general tonification/strengthening) 4. Digestive: Colic (ext-bath) 5. Neurological: epilepsy (ext-bath)	Roots rhizome, flowers	71.42	[74, 75, 84, 86]	n.y.s.
49.	*Ficus carica* L.	Moraceae	Fig tree	Smochin	Non-native	1. Blood and lymph nodes: Scrophulosis (ext-cataplasm + int- milk decoction)	Fruits	14.28	[84]	p.e.*atopic dermatitis (safety, efficacy, tolerability, and symptom relief considerable in comparison with hydrocortisone 1.0%) [115]
50.	*Filipendula ulmaria* (L.) Maxim.	Rosaceae	Mead wort	Creţuşcă	Native	1. General: Weakness (ext-bath for general tonification/strengthening)	Aerial parts	14.28	[75]	n.y.s.
51.	*Foeniculum vulgare* Mill.	Apiaceae	Fennel	Fenicul	Non-native	1. Digestive: Intestinal cramps, Flatulence	Fruits	28.57	[75, 84, 86]	p.e. infantile colic (decreased intensity of colic, decreased average daily crying time [116])
52.	*Fraxinus ornus* L.	Oleaceae	South European flowering ash	Mojdrean	Native	1. Digestive: Constipation	Sap	14.28	[75]	n.y.s.
53.	*Galium odoratum* (L.) Scop.	Rubiaceae	Sweetscented bedstraw, woodruff	Sânziene de pădure, vinariţă, muma pădurii	Native	1. Psychological (ext-bath): Fright, Bedwetting/Enuresis, Weeping during sleep 2. General: Weakness (ext-bath for general strengthening)	Aerial parts	28.57	[75, 84, 86]	n.y.s.
54.	*Galium intermedium* Schult.	Rubiaceae	-	Samca, sămcuţa, cucută de pădure	Native	1. Neurological: epilepsy (ext-bath, int- small amount of decoction)	Not specified (probably aerial parts)	14.28	[86]	n.y.s
55.	*Galium verum* L.	Rubiaceae	Lady’s bedstraw	Sânziene, drăgaică	Native	1. General: Asthenia, Weakness	Aerial parts	14.28	[75, 84]	n.y.s.
56.	*Geranium macrorrhizum* L.	Geraniaceae	Bigroot geranium	Priboi	Native	1. Neurological: Epilepsy (ext-bath)	Aerial parts, leaves	14.28	[75]	n.y.s.
57.	*Gratiola officinalis* L.	Plantaginaceae	Gratiole	Veninariţă, avramească	Native	1. Skin: Eczema 2. Nutritional: Physical debility (ext.) 3. General (fumigations): general strengthening against diseases 4. Psychological (fumigations): Fright during sleep 5. Neurological (fumigations): Epilepsy, Paralysis 6. Cardiovascular: Tahicardia	Aerial parts	85.71	[75, 85]	n.y.s.
58.	*Helianthus annuus* L.	Asteraceae	Common sunflower	Floarea soarelui	Non-native	1. Digestive: Stomach pain	Flowers, Seeds oil	14.28	[81]	n.y.s.
59.	*Heracleum sphondylium* L.	Apiaceae	Hogweed	Brânca ursului	Native	1. Psychological (ext- decoction poured on the head): Fright 2. Neurological (ext-bath): Paralysis (inability to walk)	Leaves, stems	28.57	[75]	n.y.s.
60.	1.1.1. *Elaeagnus rhamnoides* (L.) A. Nelson	1.1.2. Elaeagnaceae	Sea buckthorn	Cătină albă	Native	1. General: Weakness	Fruits	14.28	[11]	n.y.s.
61.	1.1.3. *Hordeum vulgare* L.	1.1.4. Poaceae	Barley	Ovăz	Non-native	1. General: Weakness (ext-bath)	Seeds	14.28	[84]	n.y.s.
62.	*Humulus lupulus* L.	Cannabaceae	Common hop	Hamei	Native	1. Skin: Infections, Wounds (ext-bath) 2. Nutritional: Physical debility, Underweight (ext-bath)	Flowers, leaves	28.57	[75, 84, 86]	n.y.s.
63.	*Hyoscyamus niger* L.	Solanaceae	Henbane	Măselariţă	Native	1. Psychological: Insomnia (ext-bath)	Seeds	14.28	[75]	n.y.s.
64.	*Hypericum perforatum* L.	Hypericaceae	Perforate St John’s wort	Sunătoare, pojarniţă	Native	1. Skin (ext): Eczema, Wounds, Impetigo/Skin infections, Burns	Aerial parts	14.28	[75]	n.y.s.
65.	*Impatiens noli-tangere* L.	Balsaminaceae	Touch-me-not balsam	Slăbănog	Native	1. General: weakness (ext-bath for general tonification/strengthening) 2. Musculoskeletal: Disability/Weakness of the extremities (ext-bath for bone strengthening)	Aerial parts	28.57	[75, 84, 86]	n.y.s.
66.	*Inula helenium* L.	Asteraceae	Elecampane	Iarbă mare	Native	1. Psychological: Weeping during night (ext-fumigation) 2. Neurological: epilepsy (ext-polyherbal ointment, in combination with lovage, garlic, and other plants with unresolved botanical identity)	Root	28.57	[74, 82, 86]	n.y.s.
67.	*Iris × germanica* L.#	Iridaceae	Iris	Stânjenel albastru	Native	1. Digestive: Tooth eruption pain	Rhizome	14.28	[75]	n.y.s.
68.	*Jacobaea erratica* (Bertol.) Fourr.	Asteraceae	-	Iarba carelor	Native	1. Musculoskeletal: Disability/weakness of the extremities (ext-bath for bone strengthening, in combination with water horsetail)	Not specified	14.28	[75, 79]	n.y.s.
69.	*Juglans regia* L.	Juglandaceae	Walnut tree	Nuc	Native	1. General: Weakness (int and ext- bath—for general tonification/strengthening) 2. Skin (ext): Eczema, Wounds, Skin infections ( impetigo, scabies) 3. Digestive (int- tea of shells): Diarrhea, Vomiting, Intestinal pain, Stomatitis, Intestinal parasites 4. Blood and lymph nodes: Scrophulosis (ext-bath and int), anemia 5. Nutritional: underweight (as food, in combination with bread)	Seeds, leaves, young seed shells, buds	71.42	[74, 75, 80, 84, 86, 88]	n.y.s.
70.	*Laserpitium prutenicum* L.	Apiaceae	-Prussian sermountain	Somnoroasă	Native	1. Psychological: Insomnia (ext-bath) 2. Respiratory (ext-bath): Cold, Coryza	Flowery stems	28.57	[75]	n.y.s.
71.	*Leonurus cardiaca* L.	Lamiaceae	Motherwort	Talpa gâştei, alion, somnişor	Native	1. Psychological: Insomnia (ext. bath).	Aerial parts	14.28	[85]	n.y.s.
72.	*Lepidium ruderale* L.	Brassicaceae	Peppergrass	Păduchelniţă, buruiană de roşte	Native	1. Skin: Scalp eczema, newborn scalp eczema	Aerial parts	14.28	[75, 80]	n.y.s.
73.	*Levisticum officinale* W.D.J.Koch	Apiaceae	Lovage	Leuştean	Non-native	1. Neurological: Epilepsy (ext- polyherbal ointment, in combination with *Inula helenium* L., and other plants with unresolved botanical identity)	Not specified (probably Leaves or Root)	14.28	[86]	n.y.s.
74.	*Ligustrum vulgare* L.	Oleaceae	Wild privet	Lemn câinesc	Native	1. Skin: Scabies	Bark	14.28	[74]	n.y.s.
75.	*Linaria vulgaris* Mill.	Plantaginaceae	Common toadflax	Linariţă, colţul lupului	Native	1. Skin: Subcutaneous tumors (ext)	Aerial parts	14.28	[74]	n.y.s.
76.	*Linum hirsutum* L.	Linaceae	Downy flax	Inişor de deal	Native	1. Digestive: Intestinal colics	Not specified	14.28	[75]	n.y.s.
77.	*Lycium barbarum* L.	Solanaceae	European goji	Liţion, cătină de garduri, licia	Native	1. Psychological (ext-bath): Fright/Anxiety 2. Neurological (ext-bath): Epilepsy, Spams	Aerial parts, twigs, leaves	28.57	[75, 86]	n.y.s.
78.	*Lycopodium clavatum* L.	Lycopodiaceae	Commom club moss	Brădişor, pedicuţă, coada-celor-din-vânt	Native	1. Skin (ext): Dermatitis, Eczema	Spores	14.28	[75, 84]	n.y.s.
79.	*Lysimachia nummularia* L.	Primulaceae	Moneywort	Dreţe	Native	1. General: Weakness (ext-bath for general tonification/strengthening) 2. Endocrine, metabolic, nutritional: Growth delay 3. Musculoskeletal: Disability/Weakness of the extremities (ext-bath for bone strengthening)	Aerial parts	42.85	[75, 79]	n.y.s.
80.	*Lythrum salicaria* L.	Lythraceae	Purple loosestrife	Răchitan	Native	1. Endocrine, metabolic, nutritional: underweight (ext-bath) 2. Psychological: Insomnia (int) 3. Blood and lymph nodes: anemia (int)	Aerial parts	42.85	[75]	n.y.s.
81.	*Malva sylvestris* L.	Malvaceae	Common mallow	Nalbă de pădure	Native	1. Skin: Impetigo 2. Respiratory: Tonsillitis, Diphtheric tonsillitis (int and ext), cough	Leaves, Flowers	28.57	[74, 86]	n.y.s.
82.	*Matricaria chamomilla* L.	Asteraceae	Chamomille	Museţel, Romaniță	Native	1. Digestive (int): Abdominal cramps/pains, cramps in newborns due to accumulation of gas 2. Respiratory (int): cold 3. Skin (ext): Wounds, Impetigo 4. General: pain (unspecified), bath for general strengthening of newborns, tea for internal purification (int) 5. Neurological: Epilepsy (int)	Flowers	71.42	[9, 74, 75, 86]	p.e.infantile colic (significantly more effective than simethicone [117], decreased average daily crying time [116])
83.	*Melissa officinalis* L.	Lamiaceae	Lemon balm	Roiniţă, matocină	Native	1. Neurological: Epilepsy (ext-bath)	Leaves	14.28	[86]	p.e.* infantile colic (significantly more effective than simethicone [117], decreased average daily crying time [116])
84.	*Mentha × piperita* L.#	Lamiaceae	Peppermint	Mentă, izmă bună	Native	1. Digestive tract diseases: Diarrhea, Intestinal cramps (int and ext) 2. Skin (ext): Wounds, Impetigo 3. General: ext- bath for general strengthening of newborns	Aerial parts, Leaves	42.85	[74, 75, 82, 86]	n.y.s.
85.	*Mentha pulegium* L.	Lamiaceae	Pennyroyal	Apărătoare, busuiocul cerbilor	Native	1. Endocrine, metabolic and nutritional: Underweight child/Growth dysfunctions (ext)	Aerial parts	14.28	[85]	n.y.s.
86.	*Mentha spicata* L.	Lamiaceae	Wrinkled-leaf mint	Izmă creaţă	Native	1. Digestive: Stomachache, Cramps (ext)	Leaves	14.28	[75, 83]	n.y.s.
87.	*Morus nigra* L.	Moraceae	Black mulberry	Dud negru	Non-native	1. Respiratory diseases	Fruits	14.28	[75, 86]	n.y.s.
88.	*Nepeta cataria* L.	Lamiaceae	Catnip	Cătuşnică	Native	1. Digestive: Abdominal cramps, Teeth pain, dysenteria (ext) 2. Nutritional: underweight (ext-bath) 3. Neurological: epilepsy (ext-bath), insomnia (ext-bath) 4. Musculoskeletal: bone deformities (ext-bath)	Flowers	57.14	[74, 75, 84–86]	n.y.s.
89.	*Origanum majorana* L.	Lamiaceae	Sweet marjoran	Măghiran	Non-native	1. Psychological: Weeping/Irritability	Aerial parts	14.28	[75]	n.y.s.
90.	*Origanum vulgare* L.	Lamiaceae	Oregano	Şovârf	Non-native	1. Digestive: Diarrhea, Intestinal colic (ext-bath)	Aerial parts	14.28	[74, 75, 86]	n.y.s.
91.	*Panicum miliaceum* L.	Poaceae	White millet	Mei	Non-native	1. Digestive: Digestive troubles caused by tooth eruption	Seeds	14.28	[75]	n.y.s.
92.	*Papaver rhoeas* L.	Papaveraceae	Common poppy	Mac de câmp	Native	1. General: Measles, Scarlet fever (to accelerate eruption);	Flowers	14.28	[75, 86]	n.y.s.
93.	*Papaver somniferum* L.	Papaveraceae	Opium poppy	Mac, Mac de grădină	Non-native	1. Psychological: Insomnia (int- tea or ext-bath) 2. General: Measles (int) 3. Digestive: Stomach pain, Colic 4. Respiratory: Cough (int)	Whole plant (only ext-bath), seeds	57.14	[10, 75, 80, 84, 86]	n.y.s.
94.	*Peucedanum oreoselinum* (L.) Moench	Apiaceae	Mountain parsley	Pătrunjel de câmp, Somnuroasă	Native	1. Psychological (ext-bath): Irritability, Fright, Insomnia	Flowers	14.28	[75, 78, 86]	n.y.s.
95.	*Phaseolus vulgaris* L.	Fabaceae	Common bean	Fasole	Non-native	1. Skin: Eczema, Impetigo (ext)	Seeds	14.28	[74, 75, 85, 86]	n.y.s.
96.	*Physalis alkekengi* L.	Solanaceae	Bladder cherry	Păpălău	Native	1. Skin (ext): Newborn eczema, Newborn skin eruptions	Fruits	14.28	[75]	n.y.s.
97.	*Phytolacca americana* L.	Phytolaccaceae	Pokeweed	Cârmâz	Non-native	1. General: Measles (ext. +/- int.), Scarlet fever (ext. and int.);	Fruits	14.28	[75, 84, 85]	n.y.s.
98.	*Pilosella officinarum* Vaill.	Asteraceae	mouse-ear hawkweed	Culcuşul vacii	Native	1. Neurological: Epilepsy 2. Respiratory: Cold	Aerial parts	28.57	[85]	n.y.s.
99.	*Pimpinella anisum* L.	Apiaceae	Anise	Anason	Non-native	1. Digestive: Intestinal cramps, newborn cramps due to accumulation of gas (int)	Seeds	14.28	[9, 74, 85, 86]	n.y.s.
100.	*Pinus sylvestris* L.	Pinaceae	Scots pine	Pin de pădure	Native	1. Musculoskeletal: Rickets (ext-cataplasm)	Leaves, Stems	14.28	[75]	n.y.s.
101.	*Pisum sativum* L.	Fabaceae	Pea	Mazăre, Mazerea	Non-native	1. Skin: Impetigo (ext- ash of pea)	Seeds	14.28	[86]	n.y.s.
102.	*Plantago major* L.	Plantaginaceae	Broadleaf plantain	Pătlagină mare	Native	1. Respiratory diseases (int): Cough, Whooping cough 2. Urological (int): Anuria/Oliguria 3. Digestive (int): Intestinal worms 4. Musculoskeletal: Trauma (ext) 5. General: Weakness (ext- bath for general tonification/strengthening, root, in combination with nut shell, greater celandine and musk thirstle)	Leaves, Root	71.42	[75, 86]	n.y.s.
103.	*Polypodium vulgare* L.	Polypodiaceae	Common polypody	Iarbă dulce, rădăcină dulce, feriguţă	Non-native	1. Digestive: stomachache	Rhizomes	14.28	[78]	n.y.s.
104.	*Populus spp. (P. alba* L.*, P. nigra* L.*, P. tremula* L.*)*	Salicaceae	Poplar	Plop	Native	1. General: Weakness (ext-bath)	Buds	14.28	[74]	n.y.s.
105.	*Portulaca oleracea* L.	Portulacaceae	Common purslane	Iarbă grasă	Non-native	1. General: Weakness, sickness in general (ext-bath), consumption 2. Nutritional: underweight (ext- bath for gaining weight)	Aerial parts	14.28	[80]	n.y.s.
106.	*Potentilla anserina* L. or *Argentina anserina* (L.) Rydb.§	1.1.5. Rosaceae	Silverweed	Coada racului, Scrântitoare	Native	1. General: Weakness (ext-bath for general strengthening/tonification) 2. Musculoskeletal: Rickets (ext-bath for bone strengthening)	Aerial parts	14.28	[75]	n.y.s.
107.	*Primula veris* L.	Primulaceae	Cowslip	Ciuboţica cucului	Native	1. Skin: Infections/Impetigo (ext- roots fried in butter, and boiled in milk)	Roots	28.57	[75, 84, 85]	n.y.s.
108.	*Prunella vulgaris* L.	Lamiaceae	Common self-heal	Busuioc de câmp	Native	1. Skin (ext): Wounds, Subcutaneous tumors (*lupare*)	Leaves	14.28	[75]	n.y.s.
109.	*Prunus spinosa* L.	Rosaceae	Blackthorn	Porumbar, Porumbel	Native	1. Respiratory (int): cough 2. Digestive (int): Diarrhea, Dysentery	Fruits	28.57	[86]	n.y.s.
110.	*Pulmonaria officinalis* L.	Boraginaceae	Lungworth	Mierea ursului, Plămânărică, Moşnegei	Native	1. Nutritional: Underweight (ext-bath) 2. Respiratory: scrophulosis (int- decoction)	Flowers	28.57	[74, 86]	n.y.s.
111.	*Pyrus communis* L.	Rosaceae	Pear tree	Păr	Non-native	1. Digestive (int- tea of bark): Diarrhea, vomititing 2. Nutritional: Athrepsia, Cashexia (ext- bath, leaves)	Bark, Leaves	28.57	[75, 84]	n.y.s.
112.	*Pyrus communis subsp. communis*	Rosaceae	European wild pear	Păr sălbatic	Native	1. Digestive (int- tea of bark): Diarrhea, vomititing 2. Nutritional: athrepsia, cashexia (ext- bath, leaves)	Bark, Leaves	28.57	[75]	n.y.s.
113.	*Quercus robur* L.	Fagaceae	Oak	Stejar	Native	1. Digestive: Diarrhea (int- roasted and grinded acorns)	Acorn	14.28	[75, 86]	n.y.s.
114.	*Reseda odorata* L.	Resedaceae	Garden mignonette	Rezedă, smeurică	Native	1. General: Weakness	Aerial parts	14.28	[75]	n.y.s.
115.	*Rhamnus cathartica* L.	Rhamnaceae	Common buckthorn	Verigariu	Native	1. Nutritional: athrepsia (ext-bath)	Fruits	14.28	[74, 75]	n.y.s.
116.	*Rhinanthus glaber* Lam. or *Rhinanthus angustifolius C. C. Gmel.*§	1.1.7. Orobanchaceae	Rattle	Clocotiş	Native	1. Skin: eczema (ext-bath)	Aerial parts	14.28	[75]	n.y.s.
117.	*Rhododendron myrtifolium* Schott & Kotschy	1.1.9. Ericaceae	Rhododendron	Bujor de munte, smirdar	Native	1. Skin (ext): impetigo, skin infections	Flowers, Leaves	14.28	[75]	n.y.s.
118.	*Ribes nigrum* L.	Grossulariaceae	Blackcurrant	Coacăz negru	Native	1. Nutritional: athrepsia (ext-bath)	Fruits, Leaves	14.28	[86]	n.y.s.
119.	*Robinia pseudoacacia* L.	Fabaceae	Black locust	Salcâm	Native	1. Digestive	Flowers	14.28	[87]	n.y.s.
120.	*Rosa canina* L.	Rosaceae	Dog rose	Măceş,Măcieş, Trandafir de câmp, Trandafir sălbatic, Rug	Native	1. Digestive: Diarrhea, Constipation, Colic, Abddominal pain (int-fruits, decoction, ext-flowers, decoction in vinegar) 2. Endocrine, metabolic and nutritional: lack of appetite, cachexia	Fruits, Flowers	28.57	[75, 84, 86, 87]	n.y.s.
121.	*Rubus plicatus* Weihe & Nees	Rosaceae	European blackberry	Mur negru	Native	1. Skin: Eczema (ext-ointment with burnt leaves)	Leaves	42.85	[75]	n.y.s.
122.	*Ruta graveolens* L.	Rutaceae	Rue	Rută, Virnanţ	Non-native	1. General: Fever; 2. Respiratory: Phlegm in the throat 3. Digestive: aphthous stomatitis (ext), dysenteria (int- 3 drops of juice of raw plant)	Aerial parts, Leaves	42.85	[75, 84, 86]	n.y.s.
123.	*Salix alba* L.	Salicaceae	White willow	Salcie albă, răchită	Native	1. Psychological: fright (ext-fumigation) 2. Neurological: epilepsy (int and ext-bath);	Leaves, Bark, Buds	28.57	[11, 86]	n.y.s.
124.	*Salix caprea* L.	Salicaceae	Goat willow	Iovă, Răchită moale	Native	1. General: ext- bath for general strengthening	Leaves	14.28	[75, 80]	n.y.s.
125.	*Salvia glutinosa* L.	Lamiaceae	Sticky sage	Cinsteț	Native	1. General: physical debility/weakness caused by chronic diseases	Leaves	14.28	[85]	n.y.s.
126.	*Salvia officinalis* L.	Lamiaceae	Sage	Salvie	Native	1. Respiratory diseases 2. General: Weakness (ext-bath for general strengthening)	Leaves	28.57	[75, 84]	p.e. respiratory diseases (Note. Mixed group of subjects, at least 12 year old) [118]
127.	*Sambucus ebulus* L.	Adoxaceae	Danewort	Boz	Native	1. Nutritional: underweight (ext.-bath) 2. Digestive: Cramps (int-tea), Parasites (int-decoction)	Leaves	28.57	[74, 75]	n.y.s.
128.	*Sanguisorba officinalis* L.	Rosaceae	Great burnet	Sorbestrea, Cârligăţică	Native	1. Digestive (int): Dysentery, Diarrhea, Colic 2. General: physical debility/weakness (ext. baths)	Aerial parts	28.57	[75, 85]	n.y.s
129.	*Satureja hortensis* L.	Lamiaceae	Summer savory	Cimbru de grădină	Non-native	1. Digestive (int and ext): Colic, Diarrhea 2. Blood: Anaemia 3. Skin: Eruptions, Impetigo	Aerial parts, roots, leaves, stems	42.85	[75, 85]	n.y.s.
130.	*Scabiosa columbaria* L.	Caprifoliaceae	Small scabious	Muşcatu-dracului	Native	1. General: ext-bath for newborns for general strengthening	Not specified	14.28	[75, 84]	n.y.s.
131.	*Secale cereale* L.	Poaceae	Secale	Secară	Non-native	1. General: Weakness (ext.-bath) 2. Digestive: Intestinal parasites	Seeds	28.57	[74, 86]	p.e.* improvement of cardiometabolic profile [119]
132.	*Sisymbrium officinale* (L.) Scop.	Brassicaceae	Hedge mustard	Brâncuţă, Sămcuţă	Native	1. Digestive (int): Intestinal cramps 2. Neurological (int): Epilepsy	Aerial parts	28.57	[75, 86]	n.y.s.
133.	*Solidago virgaurea* L.	Asteraceae	European goldenrod	Splinuţă, Silimină	Native	1. Musculoskeletal: Rickets (ext-bath)	Not specified (probably aerial parts)	14.28	[86]	n.y.s.
134.	*Sonchus oleraceus (*L.) L.	Asteraceae	Common sowthistle	Pălămidă grasă, Susai	Native	1. Nutritional: Athrepsia (ext-bath)	Not specified	14.28	[74]	n.y.s.
135.	*Symphytum officinale* L.	Boraginaceae	Common comfrey	Tătăneasă	Native	1. Digestive: gastro-enteritis (ext)	Root	14.28	[82]	n.y.s
136.	*Tanacetum balsamita* L.	Asteraceae	Smellin-blades	Calapăr, Calaper, Caramfil, Caromfil, Caranhil	Native	1. General: Weakness (ext- bath for general tonification/strengthening)	Aerial parts, leaves	14.28	[75, 84]	n.y.s.
137.	*Tanacetum vulgare* L.	Asteraceae	Tansy	Vetrice, măruncă	Native	1. Neurological: Epilepsy (ext-bath) 2. Skin: eczema (ext-bath)	Aerial parts	28.57	[75, 83, 86]	n.y.s.
138.	*Thymus serpyllum* L.	Lamiaceae	Wild thyme	Cimbrişor de câmp	Native	1. Digestive: Newborn cramps, flatulence	Aerial parts	14.28	[84]	n.y.s.
139.	*Thymus vulgaris* L.	Lamiaceae	Common thyme	Cimbru	Native	1. Digestive: Cramps	Aerial parts	14.28	[74]	n.y.s.
140.	*Tilia × europaea* L.#	Malvaceae	Common linden	Tei	Native	2. General or unspecified: spasm (ext-bath)	Flowers, leaves	14.28	[88]	n.y.s.
141.	*Tilia tomentosa* Moench	Malvaceae	Silver linden	Tei argintiu, tei alb	Native	1. Skin (ext): Diaper (napkin) dermatitis	Flowers	14.28	[74]	n.y.s.
142.	*Trapa natans* L.	Lythraceae	Water caltrop	Cornaci	Native	1. Digestive: Diarrhea	Fruits	14.28	[75]	n.y.s.
143.	*Trifolium arvense* L.	Fabaceae	Rabbitfoot clover	Motocei, Cotocei Iarba somnului	Native	1. Psychological (int): Irritability, Insomnia	Aerial parts	14.28	[74, 75]	n.y.s.
144.	*Valeriana officinalis* L.	Caprifoliaceae	Valerian	Odolean, valeriană	Native	1. Digestive: Diarrhea with blood/Dysenteria (ext- cataplasm on abdomen; int- 1 teaspoon of decoction made in an earthy pot) 2. Endocrine, metabolic and nutritional: Growth dysfunctions 3. Psychological: Insomnia (ext-bath) 4. Skin: Hair complaint (ext)	Root	57.14	[84, 86]	n.y.s.
145.	*Verbena officinalis* L.	Verbenaceae	Common verbena	Verbină, sporiş, sporici, buruiana de boală	Native	1. Nutritional: Underweight (ext-bath, 3 consecutive months), weakness 2. Digestive: Digestive troubles caused by tooth eruption (ext-bath) 3. Skin: Wounds, Scabies (ext)	Aerial parts	57.14	[75, 80, 86]	n.y.s.
146.	*Veronica beccabunga* L.	Plantaginaceae	European speedwell	Bobornic	Native	1. General: Weakness (ext-bath for general strengthening) 2. Digestive: Digestive troubles caused by tooth eruption (ext-bath)	Aerial parts	28.57	[75]	n.y.s.
147.	*Viburnum opulus* L.	Adoxaceae	Guilder rose	Călin	Native	1. General: Chicken pox (ext)	Fruits	14.28	[75]	n.y.s.
148.	*Vicia faba* L.	Fabaceae	Broad bean	Bob	Non-native	1. Respiratory: Tonsillitis, Diphteric tonsillitis (ext.) 2. Digestive: diarrhea	Seeds	28.57	[74, 75]	n.y.s
149.	*Viola arvensis* Murray	Violaceae	Field pansy	Panseluţă de câmp	Native	1. Skin: Eczema, Infections/Impetigo 2. Digestive: Tooth eruption (root to chew)	Aerial parts, Roots	28.57	[75]	n.y.s.
150.	*Viola odorata* L.	Violaceae	Wood violet	Toporaş, Viorea, Viorica	Native	1. Digestive: Dental eruption (root to chew)	Roots	14.28	[75, 84, 86]	n.y.s.
151.	*Viola tricolor* L.	Violaceae	Heartsease	Trei fraţi pătaţi	Native	1. Skin: Eczema, Infections/Impetigo 2. Digestive: Tooth eruption (root to chew)	Roots, probably aerial parts also	28.57	[75, 84]	n.y.s.
152.	*Xanthium strumarium* L.	Asteraceae	Common cocklebur	Scaietele popii, Ciurlan de câmp	Native	1. General or unspecified: pain in the chest (Rom. “*strâns la piept*”) (ext- warm application with boiled plant)	Not specified	14.28	[75, 80]	n.y.s.
153.	*Zea mays* L.	Poaceae	Corn	Porumb	Non-native	1. Skin (ext): Impetigo, Scabies (cob), Diaper (napkin) dermatitis	Seeds, cob	14.28	[74, 75, 84, 86]	n.y.s.

*ERTV* ethnopediatric relative therapeutic versatility (expressed as percentage), *n*.*y*.*s* not yet studied, *n*.*e*. negative evidence, *p*.*e* positive evidence; * indication not mentioned in Romanian ethnopediatry;§- pair of two Latin names for a single plant: the first name has accepted status in PlantListDB, synonym in Euro+Med PlantBase, the second name has accepted status in Euro+Med PlantBase, synonym in PlantListDB; #- Latin name has a status of accepted Latin name in PlantListDB, but it is absent in Euro+Med PlantBase

Garlic (*Allium sativum*) (ERTV 71.42%) was used for centuries in different cultures for the treatment of many diseases and is also one of the best studied herbal remedies [144]. Several recent studies raised the possibility of revival of some of its ethnopharmacological uses (mentioned also in Romanian folk medicine), which may be beneficial in child infections [99, 100], respiratory diseases [101]. In vitro experiments revealed some further therapeutic properties (benefits in alopecia areata [102], anticancer activity [145]).

Walnut tree (*Juglans regia*, Juglandaceae) (ERTV 71.42% ) is a tree known to humankind since prehistoric times, its fruits representing an important source of vitamins and nutrients [146, 147]. In Romanian ethnomedicine, *Juglans regia* is considered a panacea, a remedy that should be used for all types of diseases [74, 75, 84, 86]. Despite its traditional value, there is an obvious scarcity of studies regarding its benefits in the pediatric population. We have identified only one randomized, multicenter study evaluating the therapeutic efficacy of a herbal extract containing walnut leaves (in mixture with other six plants) in the treatment of acute non-bacterial tonsillitis in children aged 6 to 18 years [148]. The conclusion of the study was that the extract is safe and effective. There is some scientific evidence regarding the traditional use of *J*. *regia* in eczema, skin infections, scrophulosis, intestinal parasites, but not in the pediatric population. The therapeutic efficacy in adults of various *Juglans regia* extracts was proved for certain skin diseases, such as eczema [149] and atopic dermatitis [150]. The plant also showed in vitro or in vivo anti-mycobacterial [151] and antiparasitic activity [152, 153].

Chamomille (*Matricaria chamomilla* L., Asteraceae) (ERTV 71.42%) use in pediatrics is well known all over the globe [154]. Its calming effects in child colic was demonstrated by two clinical studies, both using chamomille combined with other herbs [116, 155]. According to our findings, chamomille has been traditionally used in Romania to treat skin, respiratory, and neurological pediatric conditions, too. Concerning these diseases, international clinical studies have been performed mainly in adults. They have consistently shown positive results in the treatment of wounds [156], atopic dermatitis [157, 158], migraine [159], sleep disorders [160]. Regarding the indication in respiratory disorders, a recent study evaluated the effect of a herbal mixture including chamomille in asthmatic children during viral respiratory tract infection. According to the authors, a short course of this mixture reduced cough and nighttime awakening [161]. Nevertheless, it should be pointed out that the majority of the clinical studies evaluating chamomille’s effects are deficient in quality and design [154, 162], and therefore the level of evidence is low. The antiepileptic potential of chamomille claimed by Romanian ethnomedicine remains to be investigated.

Having equal ERTV score with walnut tree and chamomile, broadleaf plantain (*Plantago major* L., Plantaginaceae) (ERTV 71.42%) is also an old European medicinal plant that has been known for centuries, especially for its wound healing properties [163]. In Romanian ethnopediatry, the plant leaves were considered to be beneficial against cough, oliguria, intestinal worms, trauma, and debility [75, 86]. Modern studies provided some evidence for its wound healing [164, 165], anti-bronchitis [166], anti-inflammatory [167, 168], immunoenhancing [169], and antiparasitic potential [170]. On the contrary, one adult clinical study reported the lack of diuretic effect, which is claimed by various ethnomedical systems [171]. No pediatric clinical study is available until now.

Blue eryngo (*Eryngium planum*, fam. Apiaceae) (ERTV 71.42%) is less scientifically studied than the previous plants. Its traditional pediatric use in skin infections, digestive colics, weakness, nutritional imbalances, and neurological diseases has no evidence until now.

### Reflections on the names of the plants

Vernacular names of plants used in Romanian ethnomedicine are deeply rooted in Romanian language. Thus, the botanist Valeriu Butura noticed in 1935 that this aspect is noticeable even in the Romanian regions where ethnic minorities were living (e.g., the Székelys, a subgroup of the Hungarian people, living in Odorhei interwar county): “…*the genuine Romanian names do not betray any foreign influence. There are only rare cases when they* (*author note: Romanian villagers*) *have adopted the terminology of the foreigners with whom they come in daily contact...*.” [83]. Pantu noticed in 1929 that many folk names of plants are quite similar to the Latin names of the plants: fag (*Fagus sylvatica* L.), fasole (*Phaseolus vulgaris* L.), frasin (*Fraxinus excelsior* L.), pin (*Pinus* spp), trifoi (*Trifolium* spp), ulm (*Ulmus* spp.), ceapă (*Allium cepa* L*.*), corn (*Cornus mas* L*.*), cicoare (*Cichorium intybus* L.), iederă (*Hedera helix* L.), vâsc (*Viscum album* L.), mentă (*Mentha piperita* L.), urzică (*Urtica dioica* L.), etc. [172]. Since Romanian language is a Latin language, the propagation of Latinity through botanical terminology even until nowadays is not an unexpected fact [173].

Nevertheless, there are regional differences in folk plant names, sometimes caused by different spoken subdialects (rom. *graiuri*) of Romanian language (ex. Moldavian subdialect, Valachian subdialect, Transylvanian subdialect, etc.). For instance, *Chelidonium majus* L., known allover Romania by its most common folk name as *rostopască*, is also called *rostopaciu*, *rostopaşte*, *rostopaste* in Transylvania, *rostopastă* in Banat, *rostopasnă*, *rostopasnică* in Bucovina, and *rătăpască* in Biharia [86].

We encountered several ambiguous cases of folk plant names—as the corresponding taxa could not be identified, those plant names and the pertaining information and/or reference were eliminated from our database. For instance, *paparoane* was reported by G. Bujorean to be used for the treatment of measles (Rom. *bubatu al mic*), but also he mentioned that this folk name corresponds to three different taxa: *Glaucium corniculatum*, *Papaver rhoeas*, *Papaver somniferum* [74]. The use of *Papaver rhoeas* for measles was unambiguously reported by other two authors who were cited in the present paper [75, 86] (and there is certainly a linguistic affinity between “paparoane” and “*Papaver*”), but we did not add G. Bujorean as a reference for this indication of *P. rhoeas*, neither did we include *Glaucium corniculatum* L. (Curtis) in our database. Nevertheless, the trust level of information found in Bujorean’s work is high grace to its precision, since he made an exact botanical identification wherever possible, excepting the cases where the informant was not able to show or describe the plant. He marked these cases in the list with folk plant names found at the end of his book, in total about 50 such unresolved botanical identity cases.

We have also noticed a complex temporal dynamic of ethnobotanical/ethnomedical terminology in Romanian ethnopharmacology. Changes of folk names of plants and diseases occurred as well with the time: some appeared recently, others disappeared, and some remained constant through all the times. For instance, G. Bujorean reported new folk names of plants in 1936 [74], which were not found in a previous national reference work, published by Z. Pantu in 1929 [172]. Latter, A. Arvat reported more new names of plants (e.g. *buruiană de cele sfinte* for *Chelidonium majus* L.) and diseases (e.g. *bube cu răutate*) [80], when compared with those cited by G. Bujorean in 1936 [74].

Also, we estimate that some folk plant names have the tendency to disappear, especially those belonging to the following categories:

- Folk plant names signifying magic potential or sacred aspects/uses of plants, e.g., *buruiana de cele sfinte* or *buruiene sfinte* (engl. *saint weed*) (*Chelidonium majus* L.) [91].
- Folk plant names indicating diseases that were no longer treated by plants at a certain point in time, e.g., *buruiana de ceas rău* (engl. *weed for bad hour/epilepsy*) (*Tanacetum vulgare* L.) [91].
- Folk plant names that contain folk disease names no longer in use, e.g., *buruiana mătricii* (*buruiana*—weed, *mătrice—*cramps in newborns) (*Tanacetum vulgare* L.) [91].

Other scientists also noticed the occurrence of this ethnopharmacological terminology dynamics in other ethnomedical systems and proposed several potential reasons for this phenomenon:

1. Change of plant indications in time, due to traditional knowledge loss (e.g., failure of intergenerational transfer of knowledge) or advances in biomedicine (e.g., antibiotics, vaccines), which outdated some plant uses [174].
2. Medicalization of folk names (replacement of folk names by new medicalized names) [175].

### Reflections on the traditional names of diseases in Romanian ethnopediatry

Romanian folk medicine, beyond its own specific local traits, has influences from humoral medicine, magic, and Christian religion [40, 97].

**Fig. 2:**
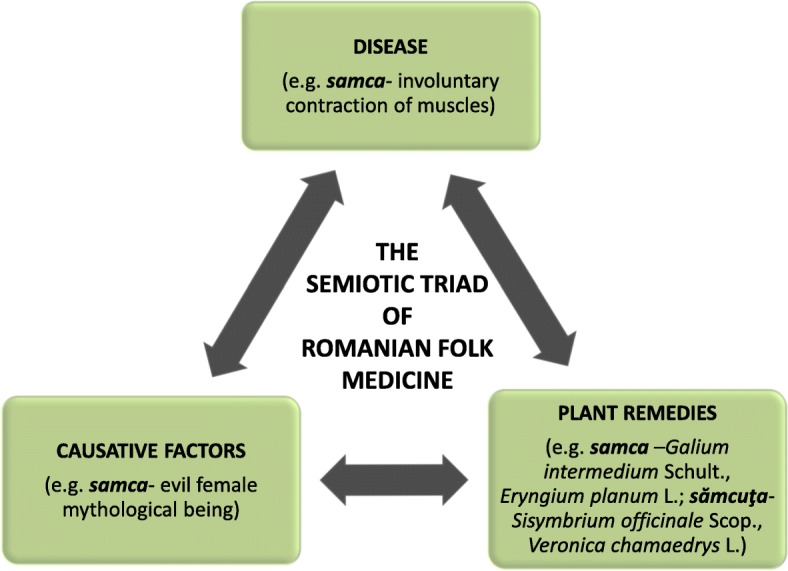
The semiotic triad of Romanian folk medicine

We have noticed that one of the specificities of the Romanian ethnomedicine is a concept that might be called “The Semiotic Triad,” which links causative factors, diseases, and therapy by theoretical and language bonds (Fig. 2). The perspective of folk Romanian medicine over the pathogenesis is that the majority of the diseases are produced by demons or unclean spirits who invade the human body (children are considered to be the most susceptible), and who often give the name of the disease, and, sometimes, even the name of the plant that represents its remedy.

For example, *samca* is an evil female mythological being (derived from Ukrainean *samka* “evil spirit, devil” or slavonic *samǔka* “woman”), also known as *aripa balaurului* (Engl. devil’s wing), who injures especially children, pregnant women, and women during postpartum period. This demon causes a disease having the same name *samca* [176]. According to the Romanian folk medicine, *samca* is a disease frequent especially among small children under four year old, characterized by tremors, spasms, involuntary contractions of muscles associated with livid colour of the body, stretching of the joints, fainting, foam at the mouth, startling during sleep, heavy sighing [84, 177–179], the description being quite similar with the clinical picture of epileptic crisis. Among the curative plants we mention, the perfect homonym plants *samca* (*Galium intermedium* Schult [86]., *Eryngium planum* L. [172]), and also the plants called *sămcuţa* (diminutive of *samca*) (*Sisymbrium officinale* Scop., *Veronica chamaedrys* L.) [86, 176]. Another example of the same kind: child nightmares, weeping during night, and consequent insomnia, a.k.a *muma*-*pădurii* are produced by *Muma-pădurii* (Engl. *Mother of the Forest*), a terrifying female spirit living in the forest, who frightens the child during the night [97], and this disturbance may be cured by *Galium odoratum* L., also called *muma pădurii* in Romanian folk medicine [91]. It is worth mentioning that this semiotic triad, which reflects the style of thought in Romanian ethnomedicine, is surprisingly more or less similar with the specificity triad proposed by Mulinari, which represents a model of a framework in the style of modern medical thought [180].

Taking into account The Semiotic Triad, it is obvious why pronouncing the name of the disease is a *tabu* in Romanian ethnomedicine, having invocation power, and at the same time, knowing the name of the causative demon offers the controlling power over the disease: the healer can throw the demon out of the body through traditional incantations (Rom. *descântece*) [97].

Certain plants with remedial strength against diseases with demon names have also folk names suggesting this ethnoiatric interdiction concerning the utterance of the disease name, for instance *Veronica chamaedrys* L. is called *tunezisă* (Engl. “you unuttered”, meaning “you with unuttered name”) [176].

Other possible ethno-ethiopathogenic factors are the charms performed by other people, divine punishments for bad actions (in case of a child, the parental bad actions), negative emotions (e.g., fright could induce *răul copiilor*- epilepsy), non-observance of certain hygienic rules (e.g., the baby breastfed by a mother with dishevelled hair would develop *bubă-*various dermatological conditions), climatic factors, such as wind, cold, heat (e.g., the child who eats snow or ice would develop *gîlci—*tonsillitis, the stroke—*luat din vânt* is caused by the wind) [97]. Some are clearly obsolete, but others may have, more or less, a scientific basis, such as lack of hygiene, cold exposure, sun exposure etc. [181, 182].

Certain folk names of diseases may reflect the ethno-ethiopathogenic factors: *boala sfântă* (Engl. saint disease)—epilepsy, *vânt rău*, *vânt sec* (Engl. bad wind, dry wind)—epilepsy, *luat din vânt* (Engl. taken from the wind)—stroke, *soare în cap* (Engl. sun in the head)—heat stroke, etc. [75, 94].

### Sacrotherapy and magic therapeutic rituals in Romanian ethnopediatry

It is worth mentioning that Romanian folk medicine, as well as ethnopediatry, is also rich in sacrotherapy habits. According to the tradition, the therapeutic efficacy of medicinal plants is increased by sanctification in the church during various religious celebrations, by hanging them at the icons, or by traditional incantations (Rom. *descântece*) [11, 79, 85, 86]. Certain magical formulas contain plants that are believed to be sacred or to bring health or luck. For instance, *număruşul* is such a magical formula transmitted in a well-known family of icon painters, living in Valea Sebeşului. The formula was worn by the child in a sachet, as an amulet, to protect him. It contained a text with the names of God in all languages, together with basil, spring wheat and frankincense [10].

### Romanian ethnopediatry and bioprospection

Analysis of our database led to the identification of several medicinal plants traditionally used in Romanian folk medicine to treat child epilepsy: *Aconitus napelus*, *Artemisia absinthium*, *Aruncus dioicus*, *Butomus umbellatus*, *Carlina acaulis*, *Centaurium erythraea*, *Cichorium intybus*, *Dryopteris filix*-*mas*, *Gratiola officinalis*, *Galium intermedium*, *Levisticum officinale*, *Lycium barbarum*, *Matricaria chamomilla*, *Melissa officinalis*, *Pilosella officinarum*, *Salix alba*, *Sisymbrium officinale*, and *Tanacetum vulgare*. Regarding these plants, their antiepileptic potential was not scientifically evaluated, except for a few studies on *Aconitum* alkaloids’ antiepileptic activity on rat hypocampal slices [183–185], one in vitro study on the inhibitory potential of *Melissa officinalis* extract on the rat brain GABA transaminase, an enzyme target in the therapy of epilepsy [186], and another one on the affinity of *Tanacetum vulgare* on the GABA(A)-benzodiazepine receptor, an alternative target in the anticonvulsant therapy [187].

Another discovery concerns the antitubercular potential of five species: *Dryopteris filix*-*mas*, *Cochlearia officinalis*, *Ficus carica*, *Juglans regia*, and *Pulmonaria officinalis L.* used to treat either tuberculosis in general or scrophulosis in Romanian ethnopediatry one century ago. This fact might be of some interest since the multidrug-resistant forms of this disease have been recently increasing in incidence in many parts of the globe [188], and new bioactive antitubercular compounds are needed.

### Prevention in Romanian ethnopediatry

We would like to highlight also that Romanian ethnopediatry has an important preventive component, several medicinal plants being used to avoid illnesses or to strengthen the body. We have found that 23 medicinal plants from a total of 153 are traditionally used as baths for general or musculoskeletal strengthening. Other examples of preventive ethnopediatry mentioned in the historical ethnographic texts are related to the management of infectious diseases: Romanian mothers used to string garlic cloves (believed to be the best remedy against *boli lipicioase*—contagious diseases) on a thread, which was afterwards attached to the baby's throat to keep him/her away from *boala de grumaz*—diphtheria [189]; the baked squash (*Cucurbita maxima*, fam. Cucurbitaceae) was applied on the child’s face in order to prevent chicken pox, especially during certain days (on the 4–6th of December, known as the “days of the chicken pox”, Rom. *zilele bubatului*) [75].

## Limitations

Our study has several limitations:

1. In certain cases, the text contained only the vernacular name of the plant, or different species had the same vernacular name. Those species whose identification credibility was very low or impossible to determine were left aside. For instance, according to Bujorean G, the vernacular name *iarba faptului* designates five species *Galium aparine*, *Sedum album*, *Geum urbanum*, *Potentilla erectum*, and *Anthyllis vulneraria* [74]. Consequently, although the plant *iarba faptului* was mentioned to be used in bath for children with skin diseases, this information was left aside.
2. We cannot claim that our data are exhaustive. The whole data on the use of medicinal plants which exists in Romanian archives has never been digitized, nor rigorously systematized. Until this process is performed, it is impossible to state that only the medicinal plants listed in this paper were historically used for children diseases on the territory of Romania in the period 1860s–1970s.
3. The number of the medicinal plants identified by us to have been used for children diseases in 1860s–1970s in Romania is an underestimation of the real number, since most probably many other plants could be indicated in child diseases, despite the fact that the ethnographers did not mentioned this. We have included in our study only the plants for which we have found the explicit specification of plant use in children. Therefore, we might have eliminated many plants widely used in children disorders, but for which this pediatric use was not specifically mentioned in our sources. This inclusion criterion was applied in order to have correct data, and to reduce to zero the risk of selecting a wrong medicinal plant. A direct question to the informant would have been necessary to establish whether a given plant used in adults was also used in children. Under these circumstances and objective constraints, we adhered to the principle that “more is not always better.” Our data might not be highly representative (“accurate”), but they are correct (“precise”).

## Conclusions

The present study on medicinal plants used in Romanian ethnopediatry exposes for the first time to the international scientific community important ethnobotanical information contained in several Romanian bibliographical resources, which are not yet translated into English, and are therefore less visible to the world experts.

Few medicinal plants (e.g., *Dryopteris filix*-*mas*, *Gratiola officinalis*, *Juglans regia*, *Eryngium planum*, *Nepeta cataria*), which were found in the present study to be significant in terms of their traditional therapeutic potential in children, are not yet (fully) studied, but their future clinical and pediatric evaluation may reveal unexpected medical benefits, especially in the case of certain diseases that still pose therapeutic challenges (e.g., epilepsy, tuberculosis). The difficulties related to pediatric clinical studies are always going to be present, due to ethical concerns, design issues (related to the continuously changing physical and biochemical features in a developing organism). Centuries-old ethnopharmacological expertise on herbal efficacy and safety could partially compensate these aspects and represent at least a starting point for further research.

The results of our search suggest that the ethnopediatric practices in Romania represent an important heritage that need to be further explored and supported in order to avail their maximum benefits. A concerted effort of botanists, pharmacologists, biochemists, and pediatric physicians is required for the assessment of available historical ethnobotanic and ethnopharmacological data. This transdisciplinary scientific analysis may lead to the discovery of new, efficient therapeutic agents for children diseases.

## Data Availability

All data generated or analyzed during this study are included in this published article.

## Abbreviations

BS: Number of targeted body systems
ERTV: Ethnopediatric relative therapeutic versatility
